# STING regulates BCR signaling in normal and malignant B cells

**DOI:** 10.1038/s41423-020-00552-0

**Published:** 2020-09-30

**Authors:** Chih-Hang Anthony Tang, Avery C. Lee, Shiun Chang, Qin Xu, Andong Shao, Yun Lo, Walker T. Spalek, Javier A. Pinilla-Ibarz, Juan R. Del Valle, Chih-Chi Andrew Hu

**Affiliations:** 1 The Wistar Institute, 3601 Spruce Street, Philadelphia, PA 19104 USA; 2 Department of Malignant Hematology, H. Lee Moffitt Cancer Center & Research Institute, Tampa, FL 33612 USA; 3 Department of Chemistry & Biochemistry, University of Notre Dame, Notre Dame, IN 46556 USA

**Keywords:** STING, BCR, ER-associated degradation, CLL, Plasma cells, Growth factor signalling, Chronic lymphocytic leukaemia, B-cell receptor

## Abstract

STING is an endoplasmic reticulum (ER)-resident protein critical for sensing cytoplasmic DNA and promoting the production of type I interferons; however, the role of STING in B cell receptor (BCR) signaling remains unclear. We generated STING V154M knock-in mice and showed that B cells carrying constitutively activated STING specifically degraded membrane-bound IgM, Igα, and Igβ via SEL1L/HRD1-mediated ER-associated degradation (ERAD). B cells with activated STING were thus less capable of responding to BCR activation by phosphorylating Igα and Syk than those without activated STING. When immunized with T-independent antigens, STING V154M mice produced significantly fewer antigen-specific plasma cells and antibodies than immunized wild-type (WT) mice. We further generated B cell-specific STING^KO^ mice and showed that STING^KO^ B cells indeed responded to activation by transducing stronger BCR signals than their STING-proficient counterparts. When B cell-specific STING^KO^ mice were T-independently immunized, they produced significantly more antigen-specific plasma cells and antibodies than immunized STING^WT^ mice. Since both human and mouse IGHV-unmutated malignant chronic lymphocytic leukemia (CLL) cells downregulated the expression of STING, we explored whether STING downregulation could contribute to the well-established robust BCR signaling phenotype in malignant CLL cells. We generated a STING-deficient CLL mouse model and showed that STING-deficient CLL cells were indeed more responsive to BCR activation than their STING-proficient counterparts. These results revealed a novel B cell-intrinsic role of STING in negatively regulating BCR signaling in both normal and malignant B cells.

## Introduction

The presence of double-stranded DNA (dsDNA) in the cytoplasm of mammalian cells is a danger signal of infection or cell anomalies. Upon binding to dsDNA in the cytoplasm, the cytoplasmic dsDNA sensor cyclic GMP-AMP synthase (cGAS) can generate 2’3’-cGAMP as an endogenous high-affinity ligand to activate stimulator of interferon genes (STING).^1–7^ STING is an endoplasmic reticulum (ER)-resident protein.^8,9^ Activation of STING leads to its translocation from the ER to the secretory pathway (i.e., the Golgi apparatus and vesicles), in which STING is phosphorylated by TANK-binding kinase 1 (TBK1), leading to the subsequent phosphorylation of interferon regulatory factor 3 (IRF3) and thus allowing for the production of type I interferons to stimulate the immune system and restore health.^8–11^ Bacteria-produced cyclic dinucleotides (e.g., c-di-AMP, c-di-GMP, and 3’3’-cGAMP) can also bind to and activate STING.^7,12–15^ STING agonists are excellent adjuvants for vaccines against viral or bacterial infections.^16,17^ STING agonists have also been proposed as combination immunotherapies with PD-1 blockers and radiation and as adjuvants to elicit potent antitumor T cell immune responses.^18–27^ These therapeutic applications of STING agonists are based on the main known function of STING, i.e., activating TBK1/IRF3 signaling to induce the production of type I interferons.

We discovered that STING agonists potently induce mitochondria-mediated apoptosis in normal and malignant B cells.^28^ Apoptosis is clearly induced through STING because no cytotoxicity is observed in STING-deficient B cell lymphoma and multiple myeloma cells. The mechanism by which activation of STING causes apoptosis of B cells remains unclear. Elucidating the differential effects of STING in B cells will be critical for successfully deploying STING agonists as therapeutic agents or vaccine adjuvants. In addition, it has been shown that the expression of STING is decreased in melanoma and colon cancer^29,30^ and that decreased levels of STING correlate with poor survival in gastric cancer patients.^31^ STING downregulation and its consequences in malignant B cells have not been investigated.

Whole-body STING^KO^ mice that were intramuscularly electroporated with a DNA vaccine encoding ovalbumin (OVA) produced significantly fewer anti-OVA antibodies than immunized wild-type (WT) mice^11^. The failure of whole-body STING^KO^ mice to mount an antibody response can result from STING deficiency in B cells, CD4 T cells, dendritic cells, or other cell types. In a recent study, B cell-specific STING^KO^ (mb-1Cre/STING^flox/flox^) mice were repeatedly immunized with OVA in combination with c-di-GMP. These immunized B cell-specific STING^KO^ mice also produced fewer anti-OVA antibodies than immunized STING^WT^ mice.^32^ Since OVA is a T-dependent antigen and c-di-GMP can still boost the type I interferon response in STING-proficient cells to influence STING-deficient B cells, it is still unclear whether STING indeed plays a role in plasma cell differentiation. Walker et al. also immunized WT and whole-body STING^KO^ mice with T-independent NP-Ficoll or NP-LPS antigen and found that the levels of anti-NP IgM were decreased only in NP-Ficoll-immunized whole-body STING^KO^ mice compared to immunized WT mice. Single immunization of B cell-specific STING^KO^ mice with a T-independent antigen in the absence of a STING agonist should allow for better elucidation of the role of STING in the formation of plasma cells.

To investigate the intrinsic function of STING in B cells, we generated a constitutively activated STING V154M knock-in mouse model and a B cell-specific STING^KO^ (CD19Cre/STING^flox/flox^) mouse model. B cells purified from STING V154M mice specifically and rapidly degraded the B cell receptor (BCR) after stimulation with lipopolysaccharide (LPS), resulting in a significant decrease in the expression of the BCR on the cell surface and reduced BCR signaling upon stimulation with goat anti-mouse IgM F(ab’)2 fragments. T-independent immunization of STING V154M mice revealed that activated STING in B cells suppressed the formation of antigen-specific plasma cells, leading to significantly decreased titers of antigen-specific antibodies. In contrast, B cells purified from B cell-specific STING^KO^ mice exhibited higher levels of the BCR on the cell surface and responded to goat anti-mouse IgM F(ab’)2 stimulation with much more robust BCR signaling than those purified from control mice. T-independent immunization of B cell-specific STING^KO^ mice confirmed that STING deficiency in B cells promoted the formation of antigen-specific plasma cells and the production of antigen-specific antibodies. Notably, both human and mouse malignant chronic lymphocytic leukemia (CLL) cells downregulated STING to promote BCR signaling for their survival.

## Results

### Generation of the STING V154M knock-in mouse model

**Fig. 1:**
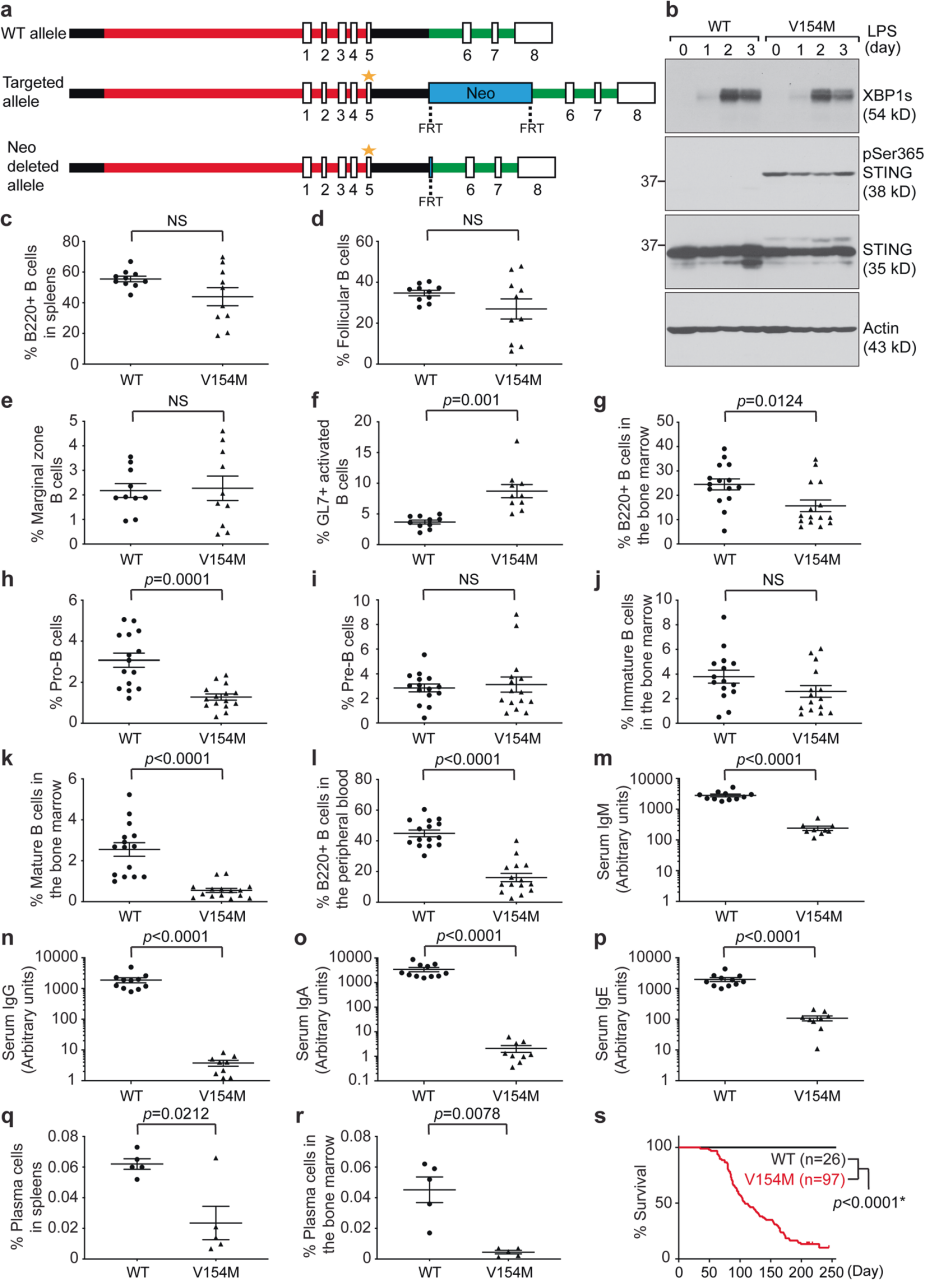
STING V154M mice exhibited defective B cell compartments and decreased levels of serum antibodies. **a** The V154 residue of mouse STING is located in exon 5. Wild-type (WT), targeted and Neo-deleted alleles are shown. The Neo cassette flanked by flippase recognition target (FRT) sites was removed by flippase (FLP) during the expansion of embryonic stem cells. V154M is indicated by the asterisk. **b** Purified WT and V154M B cells were stimulated with LPS (20 μg/mL) for 3 days. Lysates were immunoblotted for the indicated proteins. Quantification of B220+ B cells (**c**), follicular B cells (**d**), marginal zone B cells (**e**), and GL7+ activated B cells (**f**) in the spleens of unimmunized WT (*n* = 10) and V154M (*n* = 10) mice. Quantification of total B cell progenitors (**g**), pro-B cells (**h**), pre-B cells (**i**), immature B cells (**j**), and mature B cells (**k**) in the bone marrow of unimmunized WT (*n* = 15) and V154M (*n* = 15) mice. **l** Quantification of B220+ B cells in the peripheral blood of unimmunized WT (*n* = 15) and V154M (*n* = 15) mice. Serum levels of total IgM (**m**), IgG (**n**), IgA (**o**), and IgE (**p**) in unimmunized WT (*n* = 11) and V154M (*n* = 9) mice were determined by ELISA. Quantification of CD138+/XBP1s+ plasma cells in the spleens (**q**) and bone marrow (**r**) of WT (*n* = 5) and V154M (*n* = 5) mice. **s** Kaplan–Meier survival analysis of WT and V154M mice

We chose to generate STING V154M knock-in mice (Fig. 1a) because the V154M mutation leads to constitutive activation of STING.^33,34^ We performed Southern blotting to confirm the long- and short-arm integration of the targeting vector (Fig. S1) and DNA sequencing to confirm the presence of the desired mutations (GTT to ATG). Our STING V154M mouse colony was maintained by routinely crossing heterozygous V154M/WT male mice with WT/WT female mice. All experiments were carried out using 8- to 10-week-old heterozygous V154M/WT mice and their WT/WT littermates. In addition, we generated a mouse monoclonal antibody that recognized both mouse and human STING (Fig. S2A) and mapped its binding epitope (Fig. S2B–D). To detect constitutively activated mouse STING, we generated a polyclonal antibody against phospho-Ser365 of mouse STING by immunizing rabbits with a synthetic STING peptide containing the phosphorylated S365 residue. The anti-phospho-S365 antibody was affinity-purified from rabbit sera using the phosphopeptide, and then a synthetic backbone peptide was used to remove antibodies that recognized nonphosphorylated STING (Fig. S2E). Next, we purified B cells from the spleens of V154M mice and their WT littermates, stimulated these B cells with LPS to induce their differentiation into plasmablasts (as evidenced by the expression of XBP1s), and confirmed that STING was indeed constitutively phosphorylated at S365 in B cells from V154M mice (Fig. 1b).

### STING V154M mice exhibited altered B cell compartments and reduced serum antibody titers

To examine the impact of constitutively activated STING on B cell compartments, we analyzed the gated B220+ B cell population in the spleens and detected no statistically significant difference between WT and V154M mice (Figs. S3A, B and 1c). We next analyzed CD1d–/CD23+ follicular B cells and CD1d+/CD23− marginal zone B cells in the gated B220+/GL7–/AA4.1– B cell population in the spleens and found no statistically significant difference between WT and V154M mice (Figs. S3A, C, D, 1d, e). V154M mice produced more GL7+ activated B cells than their WT counterparts (Figs. S3E and 1f).

To examine B cell development in the bone marrow of V154M mice, we analyzed CD43+/CD19^low^ pro-B cells and CD43+/CD19^high^ pre-B cells in the gated B220+ B cell progenitor population (Fig. S4A). Compared with WT mice, V154M mice showed decreased percentages and numbers of total B cell progenitors and pro-B cells but not pre-B cells (Figs. 1g-i and S4B-D). We next analyzed IgM+/IgD– immature B cells and IgM+/IgD+ mature B cells in the gated CD43–/CD19+ population in the bone marrow (Fig. S4A). Compared with WT mice, V154M mice exhibited decreased percentages or numbers of immature and mature B cells (Figs. 1j-k and S4E, F). We also analyzed the gated B220+ B cell population in the peripheral blood (Fig. S4G) and detected decreases in the percentage and number of these cells in V154M mice compared to WT mice (Figs. 1l and S4H). The levels of total IgM, IgG, IgA, and IgE in the blood of V154M mice were all significantly lower than those in the blood of WT mice (Fig. 1m–p), corresponding to the decrease in the percentage of CD138+/XBP1s+ plasma cells found in the spleens and bone marrow of V154M mice (Figs. 1q-r and S4I).

### STING V154M mice exhibited altered T and myeloid cell compartments and survived shorter than their WT littermates

We next examined T cell compartments in the spleens, bone marrow, and blood of V154M mice by analyzing CD4+ and CD8+ T cells in the gated CD3+ population (Fig. S5). The percentages of both CD4+ and CD8+ T cells were found to be significantly decreased in the spleens and blood but not in the bone marrow in V154M mice compared to WT mice (Fig. S5). A significance increase in the percentage of CD4+ T cells was observed in the bone marrow of V154M mice compared to that of WT mice (Fig. S5G). We also investigated myeloid cells in the spleens, bone marrow and blood of V154M mice by analyzing Ly6C+ monocytic and Ly6G+ granulocytic cells in the gated CD11b+ population (Fig. S6). Significant increases in the percentages of both monocytic and granulocytic cells were found in the spleens, bone marrow and blood of V154M mice compared to those of WT littermates (Fig. S6). V154M mice survived significantly shorter than their WT littermates, with a median age of 108 days (Fig. 1s).

### Constitutively activated STING associated with SEL1L, HRD1, and Ig μ heavy chains, leading to rapid ERAD of the BCR in LPS-stimulated plasmablasts

**Fig. 2:**
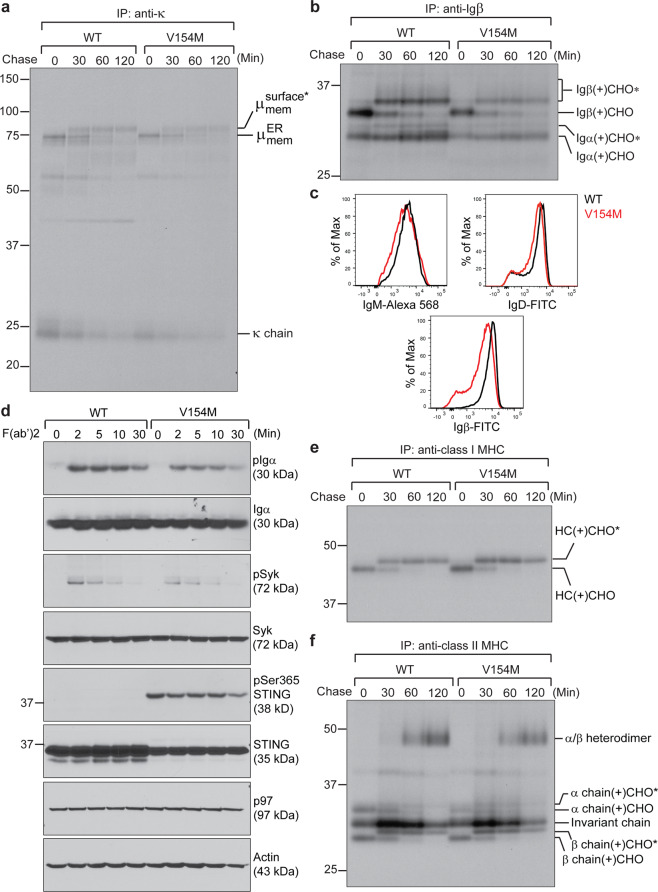
Freshly purified B cells from STING V154M mice exhibited defective surface presentation of the BCR and reduced BCR signaling upon activation. Freshly purified B cells from WT and V154M mice were starved in cysteine- and methionine-free medium for 1 h, radiolabeled for 15 min, and chased for a course of 2 h. Lysates were immunoprecipitated with an anti-κ light chain antibody (**a**) or an anti-Igβ antibody (**b**). Immunoprecipitated samples were analyzed by SDS-PAGE and autoradiography. The asterisk denotes endo‐H‐resistant complex glycans. CHO and CHO* indicate high mannose-type glycans and complex-type glycans, respectively. **c** Freshly purified B cells from WT and V154M mice were surface stained with IgM-Alexa 568, with B220-BV605 and IgD-FITC, or with CD19-Alexa 647 and Igβ-FITC. Gated live cells were analyzed for the expression of IgM (upper left panel); gated live B220+ populations were analyzed for the expression of IgD (upper right panel); and gated live CD19+ populations were analyzed for the expression of Igβ (lower panel). **d** Freshly purified B cells from WT and V154M mice were stimulated with goat anti-mouse IgM F(ab’)2 (20 μg/mL) for the indicated times and lysed for immunoblot analysis. Freshly purified B cells from WT and V154M mice were starved in cysteine- and methionine-free medium for 1 h, radiolabeled for 15 min, and chased for a course of 2 h. Lysates were immunoprecipitated with an anti-class I MHC heavy chain antibody (**e**) or an anti-class II MHC α chain antibody (**f**). Immunoprecipitated samples were analyzed by SDS-PAGE and autoradiography. CHO and CHO* indicate high mannose-type glycans and complex-type glycans, respectively

A functional BCR consists of a membrane-bound IgM (or IgD) and a disulfide-linked Igα/Igβ heterodimer. The Igα/Igβ heterodimer contains the immunoreceptor tyrosine‐based activation motifs that can be phosphorylated upon antigen binding to the membrane-bound IgM. The phosphorylated Igα/Igβ heterodimer recruits spleen tyrosine kinase (Syk), which is subsequently phosphorylated to transduce signals to promote plasma cell differentiation. To investigate how constitutively activated STING affects the BCR, we radiolabeled freshly purified splenic B cells from WT and V154M mice for 15 min and performed a 2-h chase experiment. The subsequent immunoprecipitations revealed that while B cells from V154M mice synthesized slightly lower levels of μ heavy chains, κ light chains, Igα and Igβ than those from WT mice, they could deliver the BCR to the cell surface, as evidenced by the acquisition of complex glycans in the trans-Golgi network (TGN) on all these molecules (Fig. 2a, b). Compared to WT B cells, B cells isolated from V154M mice displayed slightly decreased levels of BCR on the cell surface (Fig. 2c) and responded to stimulation with goat anti-mouse IgM F(ab’)2 with slightly reduced phosphorylation of Igα and Syk (Fig. 2d). B cells from V154M mice synthesized class I and class II MHC molecules and delivered them to the cell surface normally (Fig. 2e, f).

**Fig. 3:**
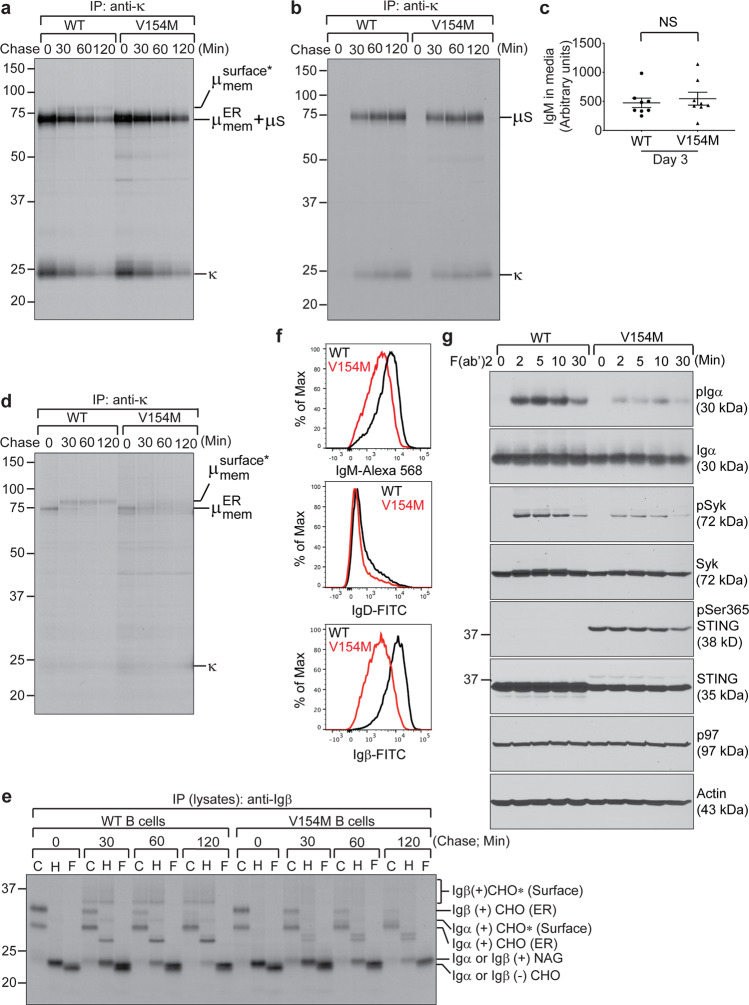
LPS-stimulated plasmablasts from STING V154M mice degraded the BCR rapidly, resulting in a significant decrease in the BCR expression on the cell surface and reduced BCR signaling upon activation. B cells from WT and V154M mice were stimulated with LPS for 3 days, starved in cysteine- and methionine-free medium for 1 h, radiolabeled for 15 min, and chased for the indicated times. Intracellular and extracellular IgM were immunoprecipitated from lysates (**a**) and culture medium (**b**), respectively, using an anti-κ antibody. The immunoprecipitates were analyzed by SDS-PAGE and autoradiography. The asterisk denotes endo‐H‐resistant complex glycans. **c** B cells purified from the spleens of WT (*n* = 8) and V154M (*n* = 8) mice were cultured at 10^6^ cells per mL in RPMI medium containing LPS (20 μg/mL) for 3 days. The levels of secretory IgM in the medium were determined by ELISA (means ± SEM). **d** B cells from WT and V154M mice were stimulated with LPS for 3 days, starved in cysteine- and methionine-free medium for 1 h, radiolabeled for 15 min, and chased for the indicated times. The cells were lysed in Triton X‐114, and the lysates were subjected to phase separation. Intracellular membrane‐bound IgM was immunoprecipitated from Triton X‐114‐associated protein fractions using an anti‐κ antibody. The asterisk denotes endo‐H‐resistant complex glycans. **e** B cells from WT and V154M mice were stimulated with LPS for 3 days, starved in cysteine- and methionine-free medium for 1 h, radiolabeled for 15 min, and chased for the indicated times. Lysates were immunoprecipitated with an anti-Igβ antibody. Immunoprecipitated Igα/Igβ heterodimers were eluted from the beads and treated with endo-H or PNGase F before analysis by SDS-PAGE and autoradiography. CHO, CHO*, and NAG indicate high mannose-type glycans, complex-type glycans and N-acetylglucosamines, respectively. **f** B cells from WT and V154M mice were stimulated with LPS for 3 days and surface stained with IgM-Alexa 568, with B220-BV605 and IgD-FITC, or with B220-BV605 and Igβ-FITC. Gated live cells were analyzed for the expression of IgM (upper panel); gated live B220+ populations were analyzed for the expression of IgD (middle panel); and gated live B220+ populations were analyzed for the expression of Igβ (lower panel). **g** B cells from WT and V154M mice were stimulated with LPS for 3 days, activated with goat anti-mouse IgM F(ab’)2 (20 μg/mL) for the indicated times and lysed for immunoblot analyses of the indicated proteins

We next exposed freshly purified B cells from WT and V154M mice to LPS for 3 days. Compared with WT B cells, V154M B cells were equally capable of differentiating into GL7+ or XBP1s+ plasmablasts (Fig. S7A-C). Surprisingly, while LPS-stimulated plasmablasts from V154M mice produced and secreted secretory IgM normally into culture media (Figs. 3a–c and S7D, E), they lacked complex glycan-modified membrane-bound μ chains (Figs. 3a and S7D). Since the ER form of the membrane-bound μ chains comigrated with secretory μ chains in SDS-PAGE gels, we phase-separated membrane-bound IgM from secretory IgM using Triton X-114.^35,36^ LPS-stimulated plasmablasts from V154M mice produced comparable levels of membrane-bound μ chains as those from WT mice, but these proteins were rapidly degraded (Fig. 3d). As a part of the BCR, Igα and Igβ in LPS-stimulated plasmablasts from V154M mice were also rapidly degraded, resulting in significantly decreased levels of complex glycan-modified Igα and Igβ in the TGN (Fig. 3e). We confirmed that the cell surface expression levels of IgM, IgD and Igβ were indeed significantly decreased in LPS-stimulated V154M plasmablasts compared to LPS-stimulated WT plasmablasts (Fig. 3f). Such reduced surface levels of the BCR led to greatly compromised BCR signaling in LPS-stimulated V154M plasmablasts upon stimulation with goat anti-mouse IgM F(ab’)2 (Fig. 3g). Notwithstanding the compromised BCR signaling, LPS-stimulated V154M plasmablasts synthesized class I and class II MHC molecules and presented them to the cell surface normally, although a slightly different glycosylation pattern was found on class II MHC molecules (Fig. S7F, G).

**Fig. 4:**
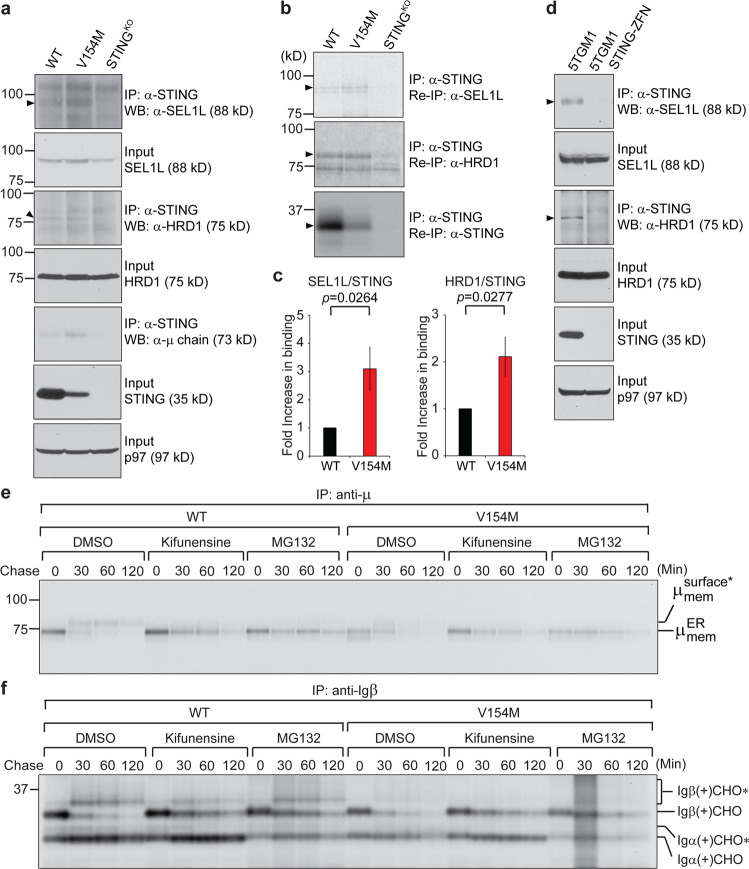
The STING V154M mutant protein increased its binding to SEL1L and HRD1 to mediate rapid degradation of the BCR. **a** B cells purified from WT, V154M and STING-deficient mice were stimulated with LPS for 3 days, lysed in 1.25% digitonin lysis buffer, and analyzed by immunoblotting for SEL1L, HRD1, STING, and p97. An anti-STING antibody was used to immunoprecipitate STING from the same lysates (equal protein amounts), and the immunoprecipitates were analyzed for the presence of SEL1L, HRD1, and Ig μ heavy chains by immunoblotting. **b** WT, V154M, and STING-deficient B cells were stimulated with LPS for 3 days, starved in cysteine- and methionine-free medium for 1 h, radiolabeled for 4 h, and lysed in 1.25% digitonin lysis buffer. The lysates were immunoprecipitated for STING, and the immunoprecipitates were subsequently boiled in 1% SDS containing 5 mM DTT and diluted with buffer containing NP-40 so that the SDS concentration was 0.05% and the NP-40 concentration was 0.5%. The diluted immunoprecipitates were divided equally into three parts, reimmunoprecipitated with an anti-STING, anti-SEL1L, or anti-HRD1 antibody, and analyzed by SDS-PAGE and autoradiography. **c** Densitometric quantitation of each radiolabeled protein band in (**b**) was achieved using phosphorimaging. The data are shown as the ratios of SEL1L/STING and HRD1/STING (means ± SEM). **d** 5TGM1 and 5TGM1 STING-ZFN (STING-deficient) cells were lysed in 1.25% digitonin lysis buffer and analyzed by immunoblotting for SEL1L, HRD1, STING and p97. An anti-STING antibody was used to immunoprecipitate STING from the same lysates (equal protein amounts), and the immunoprecipitates were analyzed for the presence of SEL1L and HRD1. **e, f** B cells from WT and V154M mice were stimulated with LPS for 3 days, starved in cysteine- and methionine-free medium for 1 h in the presence of kifunensine (50 µM) or MG132 (50 µM), radiolabeled for 15 min, and chased for 2 h in the presence of kifunensine or MG132. The cells were lysed in Triton X‐114, and the lysates were subjected to phase separation. Membrane‐bound IgM was immunoprecipitated from Triton X‐114‐associated protein fractions using an anti‐μ antibody (**e**). Igβ was directly immunoprecipitated from Triton X-114 lysates (**f**). The asterisk denotes endo‐H‐resistant complex glycans. CHO and CHO* indicate high mannose-type glycans and complex-type glycans, respectively

ER-associated degradation (ERAD) of membrane-bound μ chains has been shown to be mediated by SEL1L and the E3 ubiquitin ligase HRD1.^37,38^ We first examined the expression of SEL1L and HRD1 in WT and V154M B cells in response to LPS-stimulated differentiation and showed that both SEL1L and HRD1 were upregulated in LPS-stimulated WT and V154M plasmablasts compared to unstimulated WT and V154M B cells (Fig. S8A). We further performed immunoprecipitation using anti-STING antibodies followed by immunoblotting to detect SEL1L, HRD1 and μ chains in the immunoprecipitates (Fig. 4a). STING associated with SEL1L, HRD1 and μ chains but not with the ER-resident lectin chaperone calnexin or other type I transmembrane proteins (Figs. 4a and S8B). To quantify the increase in the interaction between the STING V154M mutant protein and SEL1L or HRD1, we radiolabeled LPS-stimulated WT and V154M plasmablasts, performed immunoprecipitations from lysates (equal counts per minute) using anti-STING antibodies, and analyzed the levels of STING, SEL1L, and HRD1 in the immunoprecipitates by reimmunoprecipitation using anti-STING, anti-SEL1L, and anti-HRD1 antibodies followed by autoradiography and quantitation by using a phosphorimager (Fig. 4b, c). The STING V154M mutant protein immunoprecipitated significantly more SEL1L and HRD1 than the WT STING protein, contributing to rapid degradation of the BCR. We further confirmed that STING could interact with SEL1L and HRD1 in STING-proficient 5TGM1 multiple myeloma cells (Figs. 4d and S8C)^28^ and that the degradation of the BCR could be blocked by kifunensine (an ERAD inhibitor) or MG132 (a proteasome inhibitor) (Fig. 4e, f).

### STING V154M mice produced significantly fewer antigen-specific plasma cells and antibodies upon T-independent immunization

**Fig. 5:**
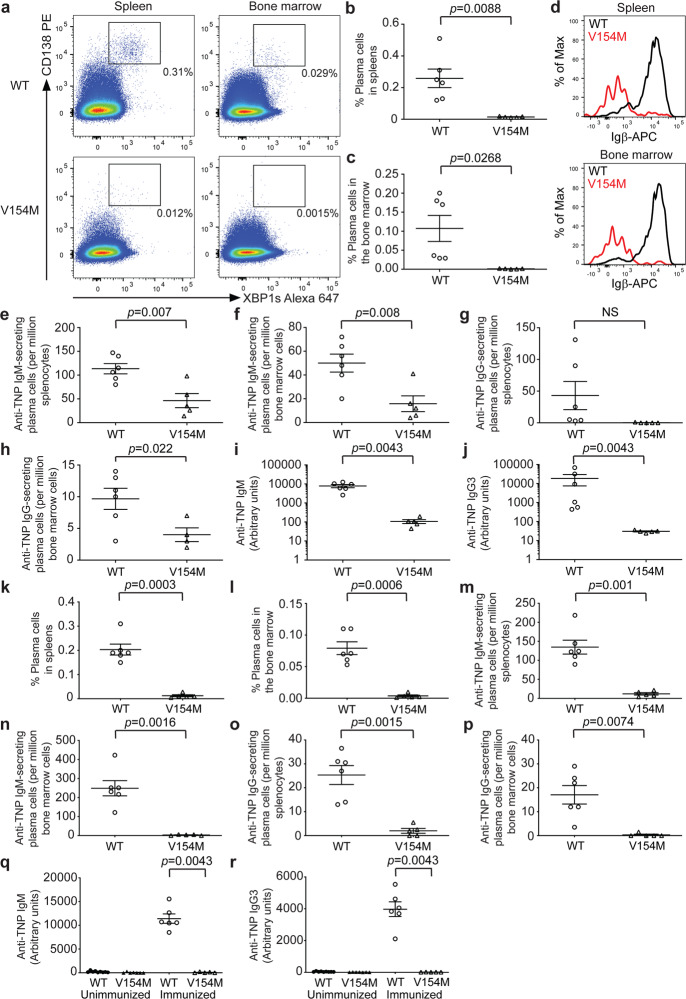
T-independently immunized V154M mice generated significantly fewer antigen-specific plasma cells and antibodies than immunized WT mice. **a** WT and V154M mice were intraperitoneally immunized with TNP-Ficoll on Day 0. On Day 9, spleen and bone marrow cells from immunized mice were stained with CD138-PE and XBP1s-Alexa 647. CD138+/XBP1s+ plasma cells were gated. Flow cytometry data from multiple WT and V154M mice were plotted as the means ± SEM and shown in (**b** and **c**). **b** Quantification of CD138+/XBP1s+ plasma cells in the spleens of TNP-Ficoll-immunized WT (*n* = 6) and V154M (*n* = 5) mice. **c** Quantification of CD138+/XBP1s+ plasma cells in the bone marrow of TNP-Ficoll-immunized WT (*n* = 6) and V154M (*n* = 5) mice. **d** WT and V154M mice were intraperitoneally immunized with TNP-Ficoll on Day 0. On Day 9, splenocytes (top panel) and bone marrow cells (bottom panel) from immunized mice were stained with B220-Alexa 488, CD138-PE and Igβ-APC. Gated B220–/CD138+ plasma cells were analyzed for the expression of Igβ. Anti-TNP IgM-secreting plasma cells in the spleens (**e**) and bone marrow (**f**) of TNP-Ficoll-immunized WT (*n* = 6) and V154M (*n* = 5) mice were quantified by ELISPOT. **g** Anti-TNP IgG-secreting plasma cells in the spleens of TNP-Ficoll-immunized WT (*n* = 6) and V154M (*n* = 5) mice were quantified by ELISPOT. **h** Anti-TNP IgG-secreting plasma cells in the bone marrow of TNP-Ficoll-immunized WT (*n* = 6) and V154M (*n* = 4) mice were quantified by ELISPOT. Nine days after the single immunization, serum levels of anti-TNP IgM (**i**) and IgG3 (**j**) in TNP-Ficoll-immunized WT (***n*** = 6) and V154M (*n* = 5) mice were determined by ELISA. **k-l** WT and V154M mice were intraperitoneally immunized with TNP-LPS on Day 0. On Day 5, spleen and bone marrow cells from immunized mice were similarly stained with CD138-PE and XBP1s-Alexa 647. CD138+/XBP1s+ plasma cells were gated. Flow cytometry data from multiple WT and V154M mice were plotted as the means ± SEM and shown in (**k** and **l**). Quantification of CD138+/XBP1s+ plasma cells in the spleens (**k**) and bone marrow (**l**) of TNP-LPS-immunized WT (*n* = 6) and V154M (*n* = 5) mice. Anti-TNP IgM-secreting plasma cells in the spleens (**m**) and bone marrow (**n**) of TNP-LPS-immunized WT (*n* = 6) and V154M (*n* = 5) mice were quantified by ELISPOT. Anti-TNP IgG-secreting plasma cells in the spleens (**o**) and bone marrow (**p**) of TNP-LPS-immunized WT (*n* = 6) and V154M (*n* = 5) mice were quantified by ELISPOT. Five days after the single immunization, serum levels of anti-TNP IgM (**q**) and IgG3 (**r**) in TNP-LPS-immunized WT (*n* = 6) and V154M (*n* = 5) mice were determined by ELISA. Sera from multiple unimmunized WT and V154M mice were used as controls

The inconsistency between the decreased antibody titers found in unimmunized V154M mice (Fig. 1m-p) and normal antibody production and secretion by LPS-stimulated V154M plasmablasts (Figs. 3a-c and Fig. S7A–E) prompted us to immunize V154M mice and investigate antigen-specific plasma cells and antibody titers. We chose to immunize WT and V154M mice with 2,4,6-trinitrophenyl (TNP)-Ficoll (a high-molecular-weight polysaccharide) because such immunization does not elicit the T cell response, allowing for a focused analysis of constitutively activated STING in B cells and plasma cells in mice. Nine days after mice were intraperitoneally injected with TNP-Ficoll, we stained both splenocytes and bone marrow cells for CD138 and XBP1s and showed that V154M mice produced significantly fewer CD138+/XBP1s+ plasma cells than WT mice in both the spleens and bone marrow (Fig. 5a–c). We further found that CD138+ plasma cells in immunized V154M mice expressed significantly lower surface levels of Igβ than those in immunized WT mice (Fig. 5d). Because B cells responded to TNP-Ficoll immunization by differentiating into TNP-specific IgM- and IgG-secreting plasma cells, we first analyzed the number of TNP-specific plasma cells by ELISPOT. TNP-Ficoll-immunized V154M mice produced significantly fewer anti-TNP IgM-secreting plasma cells in the spleens and bone marrow than immunized WT mice (Fig. 5e, f). We also detected fewer anti-TNP IgG-secreting plasma cells in both the spleens and bone marrow in immunized V154M mice than immunized WT mice, although the difference in the numbers of anti-TNP IgG-secreting plasma cells in the spleens between immunized WT and V154M mice did not reach statistical significance (Fig. 5g, h). As a result of the decrease in the numbers of anti-TNP IgM- and IgG-secreting plasma cells, V154M mice produced significantly lower serum levels of anti-TNP IgM than WT mice (Fig. 5i). Because IgG3 is the primary IgG isotype elicited by T-independent immunization, we demonstrated that anti-TNP IgG3 levels were also significantly decreased in the blood of immunized V154M mice compared with that of their immunized WT littermates (Fig. 5j).

Since V154M B cells could be stimulated by LPS in culture to differentiate into plasmablasts that produced and secreted antibodies normally (Figs. 3a–c and S7A–E), we decided to further immunize WT and V154M mice with TNP-LPS, a different T-independent antigen. Five days after the mice were intraperitoneally injected with TNP-LPS, we similarly observed significant decreases in the percentages of CD138+/XBP-1s+ plasma cells (Fig. 5k, l) and in the numbers of TNP-specific IgM- and IgG-secreting plasma cells (Fig. 5m–p) in the spleens and bone marrow of V154M mice compared to those of WT mice. TNP-LPS-immunized V154M mice produced negligible serum levels of anti-TNP IgM and IgG3 (Fig. 5q, r). These findings raised the question of whether B cell-extrinsic proinflammatory cytokines resulting from constitutive STING activation in V154M mice could influence antibody production and secretion. Thus, we further exposed LPS-stimulated WT plasmablasts to high concentrations of IFNβ or TNFα and performed similar pulse-chase experiments to answer this question. Treatment with IFNβ or TNFα did not affect antibody production or secretion by WT B cells (Fig. S9A). In addition, such treatment did not affect the surface presentation of the BCR or BCR signaling in WT B cells (Fig. S9B, C).

### Generation of a B cell-specific STING knockout mouse model

**Fig. 6:**
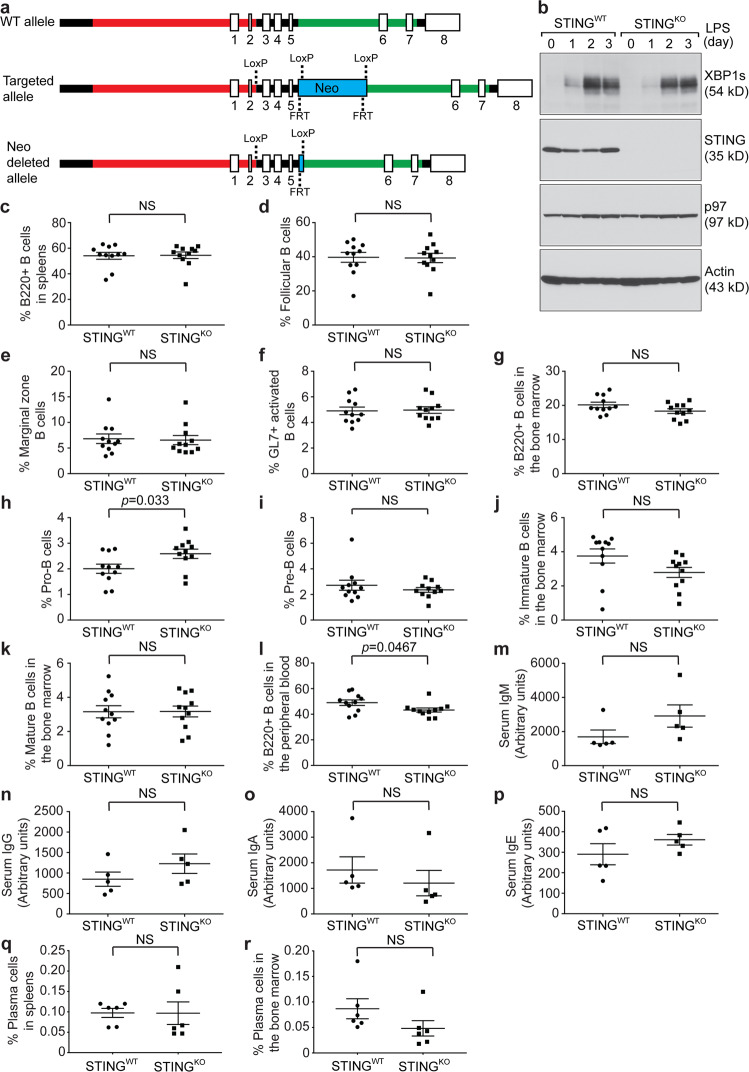
B cell-specific STING^KO^ mice exhibited nearly normal B cell compartments and serum antibody titers. **a** WT, targeted and Neo-deleted alleles are shown. Exons 3-5 of the STING gene were flanked by LoxP sites. The Neo cassette flanked by FRT sites was removed by mating chimeras carrying the successfully targeted allele with C57BL/6 FLP mice. The resultant mice carrying the Neo-deleted allele were mated with CD19Cre mice to generate B cell-specific STING^KO^ mice. **b** Purified STING^WT^ and STING^KO^ B cells were stimulated with LPS (20 μg/mL) for 3 days. Lysates were immunoblotted for the indicated proteins. Quantification of B220+ B cells (**c**), follicular B cells (**d**), marginal zone B cells (**e**), and GL7+ activated B cells (**f**) in the spleens of unimmunized STING^WT^ (*n* = 11) and STING^KO^ (*n* = 11) mice. Quantification of total B cell progenitors (**g**), pro-B cells (**h**), pre-B cells (**i**), immature B cells (**j**), and mature B cells (**k**) in the bone marrow of unimmunized STING^WT^ (*n* = 11) and STING^KO^ (*n* = 11) mice. **l** Quantification of B220+ B cells in the peripheral blood of unimmunized STING^WT^ (*n* = 11) and STING^KO^ (*n* = 11) mice. Serum levels of total IgM (**m**), IgG (**n**), IgA (**o**) and IgE (**p**) in unimmunized STING^WT^ (*n* = 5) and STING^KO^ (*n* = 5) mice were determined by ELISA. Quantification of CD138+/XBP1s+ plasma cells in the spleens (**q**) and bone marrow (**r**) of unimmunized STING^WT^ (*n* = 6) and STING^KO^ (*n* = 6) mice

To further investigate the B cell-intrinsic roles of STING in BCR activation and plasma cell differentiation, we generated a STING^flox/flox^ mouse model, in which exons 3, 4, and 5 of the STING gene were flanked by LoxP sites (Fig. 6a). Southern blotting was used to confirm the long- and short-arm integration of the targeting vector (Fig. S10). To generate a B cell-specific STING^KO^ mouse model, we crossed STING^flox/flox^ mice with CD19Cre mice, in which the CD19 promoter controls the expression of Cre recombinase. To show that B cells generated by this novel mouse model did not produce STING, we isolated B cells from the spleens of 8- to 10-week-old CD19Cre/STING^flox/flox^ (STING^KO^) and STING^flox/flox^ (STING^WT^) mice, stimulated these B cells with LPS, and confirmed the lack of expression of STING in STING^KO^ B cells (Fig. 6b).

### B cell-specific STING^KO^ mice exhibited normal B cell compartments and serum antibody titers

Using the gating strategies described in Fig. 1, we analyzed B220+ B cells, follicular B cells, marginal zone B cells, and GL7+ activated B cells in the spleens and found no difference in these populations between STING^WT^ and B cell-specific STING^KO^ mice (Fig. 6c-f). We next examined B220+ total B cell progenitors, pro-B cells, pre-B cells, immature B cells and mature B cells in the bone marrow (Fig. 6g–k) and detected a slight increase in the percentage of pro-B cells in B cell-specific STING^KO^ mice compared with STING^WT^ mice (Fig. 6h). In the peripheral blood, we detected a slight decrease in the B220+ B cell population in B cell-specific STING^KO^ mice compared with STING^WT^ mice (Fig. 6l). Nevertheless, we did not detect any significant difference in the serum levels of various antibody isotypes (Fig. 6m–p) or the percentage of plasma cells (Fig. 6q, r) between STING^WT^ and B cell-specific STING^KO^ mice.

### STING deficiency in B cells led to upregulated BCR signaling

**Fig. 7:**
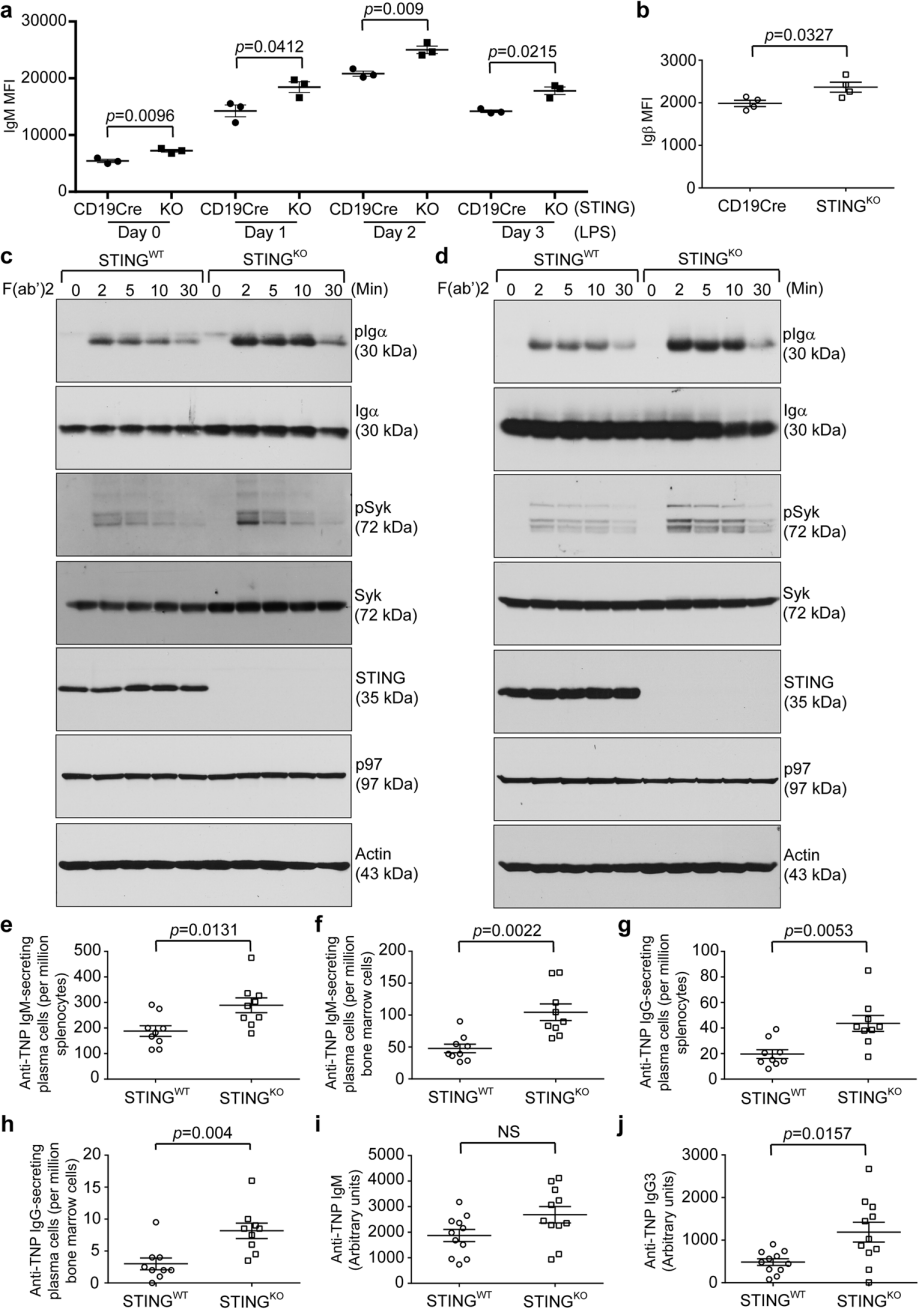
Freshly purified B cells, LPS-stimulated plasmablasts, and plasma cells from B cell-specific STING^KO^ mice exhibited significantly higher levels of surface BCR and were more responsive to BCR activation than those from STING-proficient mice; TNP-Ficoll-immunized B cell-specific STING^KO^ mice generated significantly more TNP-specific plasma cells and antibodies than TNP-Ficoll-immunized STING^WT^ mice. **a** Freshly purified B cells from CD19Cre (*n* = 3) and B cell-specific STING^KO^ (CD19Cre/STING^flox/flox^; *n* = 3) mice were stimulated with LPS (20 μg/mL) for 3 days. Each day, cells were surface stained with B220-BV605 and IgM-PE-Cy7. Gated B220+ populations were analyzed for the expression of IgM. The mean fluorescence intensity (MFI) of IgM was plotted as the mean ± SEM. **b** CD19Cre (*n* = 4) and B cell-specific STING^KO^ (*n* = 4) mice were intraperitoneally immunized with TNP-Ficoll on Day 0. On Day 9, bone marrow cells from immunized mice were stained with B220-Alexa 488, CD138-PE, and Igβ-APC. Gated B220–/CD138+ plasma cells were analyzed for the expression of Igβ. The MFI of Igβ was plotted as the mean ± SEM. **c** Freshly purified B cells from STING^WT^ and STING^KO^ mice and (**d**) those B cells stimulated with LPS for 3 days were activated with goat anti-mouse IgM F(ab’)2 (20 μg/mL) for the indicated times and lysed for immunoblot analysis. **e-j** STING^WT^ and B cell-specific STING^KO^ mice were intraperitoneally immunized with TNP-Ficoll on Day 0. Immunized mice were sacrificed on Day 9, and the presence of TNP-specific plasma cells in the spleens and bone marrow was analyzed. Anti-TNP IgM-secreting plasma cells in the spleens (**e**) and bone marrow (**f**) of TNP-Ficoll-immunized STING^WT^ (*n* = 9) and B cell-specific STING^KO^ (*n* = 9) mice were quantified by ELISPOT. Anti-TNP IgG-secreting plasma cells in the spleens (**g**) and bone marrow (**h**) of TNP-Ficoll-immunized STING^WT^ (*n* = 9) and B cell-specific STING^KO^ (*n* = 9) mice were quantified by ELISPOT. Nine days after the single immunization, serum levels of anti-TNP IgM (**i**) and IgG3 (**j**) in TNP-Ficoll-immunized STING^WT^ (*n* = 11) and B cell-specific STING^KO^ (*n* = 11) mice were determined by ELISA

To investigate the B cell-intrinsic roles of STING in the synthesis and intracellular transport of integral membrane and secretory proteins, we first performed pulse-chase experiments to examine the BCR. B cells freshly purified from CD19Cre/STING^flox/flox^ (STING^KO^) mice synthesized and delivered slightly higher levels of the BCR to the cell surface than those from STING^flox/flox^ (STING^WT^) mice (Fig. S11A-C). Since STING^KO^ B cells contained only one functional CD19 allele, we decided to also compare the surface expression levels of the BCR on B cells from STING^KO^ and CD19Cre/STING^WT/WT^ mice. Indeed, the surface expression of membrane-bound IgM was consistently higher in B cells from STING^KO^ mice than in those from CD19Cre/STING^WT/WT^ mice regardless of whether the B cells were stimulated with LPS (Figs. 7a and S11D). We also immunized STING^KO^ and CD19Cre/STING^WT/WT^ mice with TNP-Ficoll and found that CD138+ plasma cells in immunized STING^KO^ mice expressed significantly higher surface levels of Igβ than those in immunized CD19Cre/STING^WT/WT^ mice (Figs. 7b and S11E). The increased BCR levels might have resulted from the decreased levels of SEL1L, indicating reduced ERAD, in STING^KO^ plasmablasts (Fig. 4a).

Since B cells, plasmablasts, and plasma cells from STING^KO^ mice exhibited higher levels of membrane-bound IgM and Igβ on their surface than those from CD19Cre/STING^WT/WT^ mice, we next examined the impact of STING deficiency on BCR signaling by exposing freshly purified B cells and LPS-stimulated plasmablasts from STING^WT^ and STING^KO^ mice to goat anti-mouse IgM F(ab’)2. Both freshly purified STING^KO^ B cells and LPS-stimulated STING^KO^ plasmablasts responded to goat anti-mouse IgM F(ab’)2 by inducing significantly higher levels of phospho-Igα and phospho-Syk than their STING^WT^ counterparts did (Figs. 7c, d and S12A, B).

### B cell-specific STING^KO^ mice produced significantly increased numbers of antigen-specific plasma cells and titers of antigen-specific antibodies upon T-independent immunization

To investigate the B cell-intrinsic roles of STING in the generation of antigen-specific plasma cells and antibodies, we immunized STING^WT^ and B cell-specific STING^KO^ mice with TNP-Ficoll and analyzed the numbers of TNP-specific plasma cells by ELISPOT. TNP-Ficoll-immunized B cell-specific STING^KO^ mice produced significantly more anti-TNP IgM- and IgG-secreting plasma cells in the spleens and bone marrow than immunized STING^WT^ mice (Fig. 7e-h). Although we did not detect a statistically significant increase in anti-TNP IgM levels in the sera of TNP-Ficoll-immunized B cell-specific STING^KO^ mice compared to their immunized STING^WT^ counterparts (Figs. 7i and S12C), these immunized STING^KO^ mice produced significantly higher serum levels of anti-TNP IgG3 (Figs. 7j and S12D).

### CLL cells downregulated the expression of STING to promote BCR signaling

**Fig. 8:**
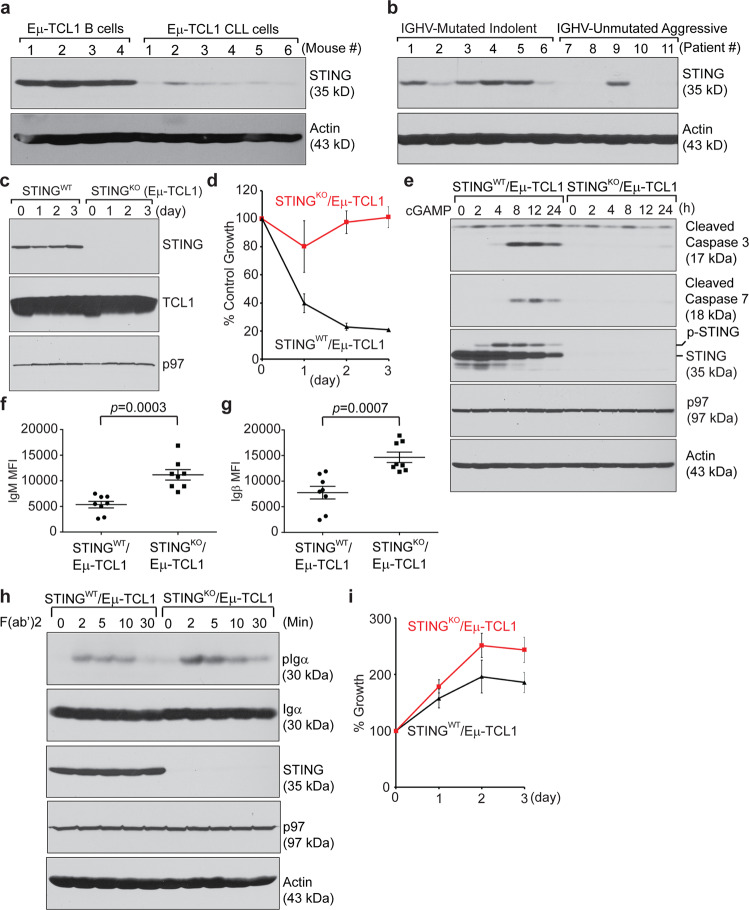
Malignant CLL cells downregulated the expression of STING to promote BCR signaling and cell proliferation. **a** B cells purified from the spleens of four 6-week-old Eμ-TCL1 mice and CLL cells purified from the spleens of six CLL-bearing Eμ-TCL1 mice were lysed and immunoblotted for the indicated proteins. **b** Six IGHV-mutated indolent (patients # 1~6) and five IGHV-unmutated aggressive (patients # 7~11) CLL samples were lysed and immunoblotted for the indicated proteins. **c** B cells purified from the spleens of STING^WT^/Eμ-TCL1 and STING^KO^/Eμ-TCL1 mice were stimulated with LPS for 3 days and immunoblotted for the indicated proteins. **d** STING^WT^/Eµ-TCL1 and STING^KO^/Eµ-TCL1 CLL cells were treated with 3’3-cGAMP (20 µM) for 3 days in the presence of LPS and subjected to XTT assays at the end of each day. The percentage of growth was determined by comparing cells treated with LPS plus cGAMP with those treated with LPS alone. Each data point derived from four independent groups receiving the same treatment was plotted as the mean ± SD. **e** Purified STING^WT^/Eµ-TCL1 and STING^KO^/Eµ-TCL1 CLL cells were stimulated with LPS for 2 days and subsequently treated with 3’3-cGAMP (20 µM) for 24 h. The cells were lysed and immunoblotted for the indicated proteins. **f** Spleen cells from STING^WT^/Eµ-TCL1 (*n* = 8) and STING^KO^/Eµ-TCL1 (*n* = 8) mice were surface stained with CD19-APC-Cy7, IgM-Alexa 568, B220-Alexa 488 and CD5-APC. B220^low^CD5+ CLL cells in the gated CD19+ B cell population were analyzed for the expression of IgM. The MFI of IgM was plotted as the mean ± SEM. **g** Spleen cells from STING^WT^/Eµ-TCL1 (*n* = 8) and STING^KO^/Eµ-TCL1 (*n* = 8) mice were surface stained with CD19-APC-Cy7, Igβ-FITC, B220-BV605, and CD5-APC. B220^low^CD5+ CLL cells in the gated CD19+ B cell population were analyzed for the expression of Igβ. The MFI of Igβ was plotted as the mean ± SEM. **h** Freshly purified STING^WT^/Eµ-TCL1 and STING^KO^/Eµ-TCL1 CLL cells were stimulated with goat anti-mouse IgM F(ab’)2 (20 µg/mL) for the indicated times and lysed for immunoblot analysis. **i** STING^WT^/Eµ-TCL1 and STING^KO^/Eµ-TCL1 CLL cells were stimulated with LPS (20 µg/mL) for 3 days. At the end of each day, the cells were subjected to XTT assays. The percentage of growth was determined by comparing LPS-treated groups with untreated (Day 0) groups. Each data point derived from four independent groups receiving the same treatment was plotted as the mean ± SD

The Eµ-TCL1 transgenic mouse model was established by placing the human TCL1 gene under the control of an immunoglobulin heavy chain promoter/enhancer to drive TCL1 overexpression in mouse B cells.^39^ This model recapitulates aggressive IGHV-unmutated human CLL with constitutive BCR signaling that supports high-rate proliferation of CLL cells.^40–42^ We purified CD19+B220^high^CD5– B cells from the spleens of 6-week-old Eμ-TCL1 mice and CD19+B220^low^CD5+ CLL cells from the spleens of CLL-bearing Eμ-TCL1 mice and showed that mouse CLL cells expressed significantly less STING than mouse B cells (Fig. 8a). Unlike untreated IGHV-mutated indolent human CLL samples, many untreated IGHV-unmutated aggressive human CLL samples produced undetectable levels of STING (Fig. 8b). To test our hypothesis that a decrease in or the loss of STING in CLL cells might lead to increased BCR signaling to support CLL growth, we generated a novel STING^KO^/Eµ-TCL1 CLL mouse model by crossing B cell-specific STING^KO^ mice with Eµ-TCL1 mice (Fig. 8c) and purified CD19+B220^low^CD5+ CLL cells from CLL-bearing STING^WT^/Eµ-TCL1 and STING^KO^/Eµ-TCL1 mice (Fig. S13A). Similar to STING-deficient A20 B cell lymphoma cells and 5TGM1 multiple myeloma cells,^28^ STING^KO^/Eµ-TCL1 CLL cells were resistant to 3’3’-cGAMP-induced apoptosis (Fig. 8d, e). Compared with STING^WT^/Eµ-TCL1 CLL cells, STING^KO^/Eµ-TCL1 CLL cells expressed higher levels of IgM and Igβ on their surface (Figs. 8f-g and S13B, C). Upon stimulation with goat anti-mouse IgM F(ab’)2, STING^KO^/Eµ-TCL1 CLL cells exhibited more robust BCR signaling than their STING^WT^/Eµ-TCL1 CLL counterparts (Fig. 8h). In response to stimulation with LPS, these STING-deficient CLL cells also proliferated significantly faster than their WT counterparts (Fig. 8i).

## Discussion

We showed that STING played a role in ERAD of the BCR by interacting with SEL1L and HRD1 (Fig. 4a–d). Compared with freshly purified V154M B cells, which could deliver the BCR to the B cell surface (Fig. 2a–c), stimulation of V154M B cells with LPS for 3 days led to rapid degradation of the BCR and impaired signal transduction of the BCR upon stimulation with F(ab’)2 (Figs. 3a and d-g and S7D). This phenomenon might have resulted from the significant increase in SEL1L and HRD1 levels in V154M plasmablasts after stimulation with LPS for 3 days. Compared to freshly purified V154M B cells, the V154M plasmablasts generated after 3-day LPS stimulation expressed comparable levels of STING (Fig. S8A). Although STING was expressed at lower levels in V154M plasmablasts than in WT plasmablasts, the mutant STING protein efficiently bound to SEL1L and HRD1 (Fig. 4a–c) and mediated faster ERAD of the BCR in V154M plasmablasts than in WT plasmablasts (Figs. 3a, d and e and S7D). Recently, STING was shown to engage TRIM21, another E3 ubiquitin ligase, to degrade γ-interferon-inducible protein-16.^43^

Our study revealed a novel B cell-intrinsic role of STING in regulating BCR signaling and plasma cell differentiation. Since continuous BCR signaling via repeated antigen binding is critical for driving plasma cell differentiation, insufficient BCR signaling in V154M B cells (Fig. 2d) and LPS-stimulated V154M plasmablasts (Fig. 3g) could explain why T-independently immunized V154M mice produced significantly decreased antigen-specific plasma cells and antibodies (Fig. 5). Consistent with these data, STING-deficient B cells and plasmablasts transduced more robust BCR signaling than their STING-proficient counterparts (Figs. 7c, d and S12A, B), accounting for the increase in the numbers of antigen-specific plasma cells accompanied by the increase in antigen-specific antibody titers in B cell-specific STING^KO^ mice after T-independent immunization (Fig. 7e–j). Importantly, our data also revealed that STING downregulation in CLL cells could contribute to upregulated BCR signaling to support the survival of CLL cells (Fig. 8). This effect is different from the proposed extrinsic role of STING downregulation in curtailing the production of type I interferons and avoiding the activation of antitumor immunity in other cancer types.

Several research groups have independently generated mouse models expressing constitutively activated STING, namely, N153S^44^ and V154M.^45^ These studies focused on analyzing immune cell populations in older mice with autoinflammatory syndrome and showed that the syndrome could not be alleviated by genetic deletion of IRF3 or IFN-α/β receptor signaling. Warner et al. also reported that 4- to 6-month-old N153S mice did not produce significantly more B cells in the spleens than WT mice; however, N153S mice produced significantly higher levels of total IgM than WT mice but similar levels of total IgG as WT mice.^44^ Bouis et al. reported that unlike N153S mice, 2- to 5-month-old V154M mice produced significantly fewer B cells in the spleens and significantly lower levels of total IgM, IgG and IgA in the blood than WT mice.^45^ Although we found that our V154M mice similarly produced lower serum levels of antibodies than WT mice (Fig. 1m–p), our results also clearly showed that B cells purified from V154M mice responded normally to LPS in culture by differentiating into plasmablasts and producing antibodies (Figs. 3a–c and S7A-E). Such discordant results prompted us to investigate whether V154M B cells could respond to T-independent immunization by differentiating into antigen-specific plasma cells and producing antigen-specific antibodies in mice. We investigated B cell populations and performed immunization in V154M mice between the ages of 8 and 10 weeks before the autoinflammatory syndrome developed. While there was no statistically significant difference in the numbers of total splenic B220+ B cells between unimmunized V154M mice and their WT counterparts (Figs. 1c and S3B), we observed significant decreases in the numbers of total B220+ B cells in the bone marrow and peripheral blood in 8- to 10-week-old V154M mice compared to WT mice (Figs. 1g and l, S4B, H). We also detected significant decreases in the percentages of CD138+/XBP1s+ plasma cells in unimmunized V154M mice compared to unimmunized WT mice (Fig. 1q, r). We observed that upon T-independent immunization, V154M mice generated significantly fewer antigen-specific plasma cells and antibodies (Fig. 5e-j and m-r). Together with our data showing that STING^KO^ B cells exhibited higher levels of the BCR on the surface than STING-proficient B cells (Figs. 7a and S11A-D) and that T-independently immunized B cell-specific STING^KO^ mice produced more antigen-specific plasma cells and antibodies than T-independently immunized STING^WT^ mice (Fig. 7e–j), we hypothesized that constitutively activated STING could efficiently engage SEL1L/HRD1-mediated ERAD of the BCR, resulting in insufficient BCR signaling, which in turn contributed to impaired plasma cell differentiation and reduced antibody production in vivo. While LPS induced V154M B cells to differentiate into plasmablasts and produce antibodies in culture, TNP-LPS did not induce V154M B cells to differentiate into TNP-specific plasma cells and produce TNP-specific antibodies in mice. We hypothesized that the significant alteration of T and myeloid cell populations (Figs. S5, S6) resulting from constitutively activated STING might contribute to suppressed plasma cell differentiation and function in V154M mice.

STING activation as a result of the V154M mutation could cause ERAD of the BCR, thus causing decreased surface presentation of the BCR and reduced BCR signaling (Figs. 3a and d-g, 4a-c, and S7D). We previously showed that activation of STING by high-affinity STING agonists, such as 3’3’-cGAMP, could lead to rapid apoptosis of B cells and plasmablasts.^28^ V154M-mediated STING activation clearly did not trigger rapid apoptosis of B cells and plasmablasts, as evidenced by the fact that freshly purified B cells carrying the STING V154M mutation produced the BCR as well as both class I and class II MHC molecules and delivered these molecules to the cell surface (Fig. 2a-c and e, f). Even when B cells from V154M mice were stimulated with LPS for 3 days in culture, they still synthesized normal levels of secretory IgM molecules and secreted them into the culture medium (Figs. 3 a–c and S7D, E). We hypothesized that STING activated by the V154M mutation may assume a conformation different from that of STING bound to a high-affinity agonist, allowing for its interaction with distinct protein partners and leading to different phenotypes in B cells.

Walker et al. generated a different B cell-specific STING^KO^ (mb-1Cre/STING^flox/flox^) mouse model, but these mice were not used for analysis of antibody response after immunization with a T-independent antigen.^32^ In studies by Walker et al., whole-body STING^KO^ mice were immunized with a T-dependent (OVA or SRBC) or T-independent (NP-Ficoll or NP-LPS) antigen. When whole-body STING^KO^ mice were immunized with a T-dependent antigen (NP-OVA), there was no change in their antibody response. Only when whole-body STING^KO^ mice were immunized with SRBC (a partly T-dependent and partly T-independent antigen) or with NP-Ficoll (a T-independent antigen) was reduced antibody production observed. Reduced antibody production was not observed in whole-body STING^KO^ mice immunized with NP-LPS. Immunization of whole-body STING^KO^ mice could involve T cells, follicular dendritic cells, and other immune cells, making it difficult to evaluate the B cell-intrinsic roles of STING. We immunized B cell-specific STING^KO^ (CD19Cre/STING^flox/flox^) mice with a T-independent antigen TNP-Ficoll and found significant increases in the numbers of antigen-specific plasma cells and antibody levels in these mice compared with their WT counterparts.

## Materials and methods

### Generation of a V154M knock-in mouse model and B cell-specific STING^KO^ mice

STING V154M knock-in and STING floxed (STING^flox/flox^) mice were generated using conventional gene targeting methods (see Figs. 1a, 6a), similar to previous studies.^46^ The presence of the point mutation was confirmed by DNA sequencing. Southern blotting analyses were used to confirm the integration of 3’ and 5’ homology arms, as described previously.^46^ Male heterozygous V154M/WT mice were routinely crossed with female WT/WT mice to generate 50% V154M/WT mice and 50% WT/WT mice. STING^flox/flox^ mice were crossed with CD19Cre (Cre recombinase driven by the CD19 promoter) mice to generate B cell-specific STING^KO^ (CD19Cre/STING^flox/flox^) mice.

### Mice

WT, STING V154M knock-in, B cell-specific STING^KO^, CD19Cre, Eμ-TCL1, STING^WT^/Eμ-TCL1, and STING^KO^/Eμ-TCL1 mice were maintained at our animal facility strictly following the guidelines provided by the Wistar Institute Animal Care and Use Committee.

### Study approval

All experiments using mice were performed following protocols approved by the Wistar Institute Animal Care and Use Committee.

### Purification of mouse B cells

Mouse B cells were purified from the mouse spleen by negative selection using CD43 (Ly48) or Pan-B magnetic beads (Miltenyi Biotec) according to the manufacturer’s instructions.

### Flow cytometry analysis

Following RBC lysis, single cell suspensions from the spleen, bone marrow or peripheral blood were blocked for 30 min using FBS. Intracellular staining of XBP-1s with an anti-XBP1s antibody conjugated to Alexa 647 (Q3-695; BD Biosciences) was achieved using the BD Pharmingen^TM^ Transcription Factor Buffer Set. We performed cell surface staining by incubating cells at 4 °C for 30 min with the following fluorescence-conjugated anti-mouse antibodies (clone; source): B220-Alexa 488 (RA3-6B2; Biolegend); B220-BV605 (RA3-6B2; Biolegend); CD43-PE (S11; Biolegend); CD19-Alexa 647 (6D5; Biolegend); CD19-APC-Cy7 (6D5; Biolegend); CD5-APC (53-7.3; Biolegend); CD3-BV605 (145-2C11; Biolegend); CD3-APC-Cy7 (145-2C11; Biolegend); CD4-BV605 (RM4-5; Biolegend); CD8-PE-Cy7 (53-6.7; Biolegend); IgM-PE-Cy7 (RMM-1; Biolegend); IgM-Alexa 568 (A-21043; Invitrogen); IgD-FITC (11-26 c.2a; Biolegend); GL7-PE (GL7; Biolegend); AA4.1-PE-Cy7 (AA4.1; Biolegend); CD1d-PerCP-Cy5.5 (1B1; Biolegend); CD23-FITC (B3B4; Biolegend); Igβ-FITC (HM79-12; Biolegend); Igβ-APC (HM79-12; Biolegend); CD138-PE (281-2; Biolegend); CD11c-BV421 (N418; Biolegend); CD11b-PE (M1/70; Biolegend); Ly6C-Alexa 488 (HK1.4; Biolegend); and Ly6G-Alexa 647 (1A8; Biolegend). Viability staining was accomplished using DAPI exclusion or Fixable Viability Stain 450 (BD Biosciences) during acquisition. Acquisition of immune cell populations was performed on an LSRII cytometer (BD Biosciences) harboring a custom configuration for the Wistar Institute. Cytometry data were analyzed using FlowJo software version 10.1 (Tree Star Inc.).

### Antibodies and reagents

We generated a mouse monoclonal antibody that recognized an epitope within amino acids 322-339 of human STING and within amino acids 321-338 of mouse STING (Fig. S2A-D). A polyclonal antibody against phospho-S365 of mouse STING was generated in rabbits and affinity-purified (Fig. S2E). Polyclonal antibodies against mouse STING, Igα, Igβ, class I MHC heavy chain, and class II MHC α chain were generated in rabbits. An anti-SEL1L polyclonal antibody was also generated in rabbits and affinity-purified. The following antibodies were purchased: XBP1s (Cell Signaling), HRD1 (Abgent), HRD1 (Proteintech), p97 (Fitzgerald), μ (SouthernBiotech), κ (SouthernBiotech), phospho-Syk (Cell Signaling), Syk (Cell Signaling), phospho-Igα (Cell Signaling), TCL1 (Cell Signaling), cleaved caspase 3 (Cell Signaling), cleaved caspase 7 (Cell Signaling), calnexin (Proteintech), and actin (Sigma). LPS and digitonin were purchased from Sigma. Recombinant mouse IFNβ and TNFα were procured from Biolegend. Kifunensine and MG132 were purchased from Cayman. 3’3’-cGAMP was chemically synthesized as described previously.^28^

### Cell culture

Purified mouse B cells, mouse A20 lymphoma B cells (ATCC), mouse A20 STING-ZFN (STING^KO^) lymphoma B cells,^28^ mouse 5TGM1 multiple myeloma cells, mouse 5TGM1 STING-ZFN (STING^KO^) multiple myeloma cells,^28^ and human H929 myeloma cells (ATCC) were cultured in RPMI 1640 medium (Gibco) supplemented with 10% heat-inactivated fetal bovine serum (FBS), 2 mM L-glutamine, 100 U/mL penicillin G sodium, 100 µg/mL streptomycin sulfate, 1 mM sodium pyruvate, 0.1 mM nonessential amino acids, and 0.1 mM β-mercaptoethanol (β-ME). All our cell lines were negative for mycoplasma contamination.

### Immunoblotting

Cells were lysed in RIPA buffer (10 mM Tris-HCl, pH 7.4; 150 mM NaCl; 1% NP-40; 0.5% sodium deoxycholate; 0.1% SDS; and 1 mM EDTA) supplemented with protease inhibitors (Roche) and phosphatase inhibitors. Protein concentrations were determined by the BCA assay (Pierce). The proteins were boiled in SDS-PAGE sample buffer (62.5 mM Tris-HCl, pH 6.8; 2% SDS; 10% glycerol; and 0.1% bromophenol blue) containing β-ME, analyzed by SDS-PAGE, and transferred to nitrocellulose membranes, which were subsequently blocked in 5% nonfat milk (wt/vol in PBS) and immunoblotted with the indicated primary antibodies and appropriate horseradish peroxidase (HRP)-conjugated secondary antibodies. The immunoblots were developed using Western Lightning Chemiluminescence Reagent (Perkin-Elmer).

### Pulse-chase experiments, immunoprecipitation, and deglycosylation

Cells were starved in methionine- and cysteine-free medium containing dialyzed FBS for 1 h and pulse-labeled with 250 μCi/mL [^35^S]-methionine and [^35^S]-cysteine (Perkin-Elmer) for 15 min. After labeling, the cells were incubated in the chase medium containing cold methionine (2.5 mM) and cysteine (0.5 mM). At the end of each chase interval, radiolabeled cells were lysed in RIPA buffer containing protease inhibitors. Radiolabeled cells were lysed in 10 mM Tris-HCl, pH 7.4, 150 mM NaCl, and 1% Triton X-114 on ice to allow for subsequent phase separation of membrane-bound IgM from secretory IgM using previously described methods.^35,36^ Precleared lysates were incubated with antibodies against Ig κ light chain, Ig μ heavy chain, Igβ, class I MHC heavy chain, or class II MHC α chain, together with protein G-sepharose beads. For deglycosylation experiments, bead-bound Igβ was eluted and denatured in glycoprotein denaturing buffer (0.5% SDS, 40 mM DTT) at 95 °C for 10 min, treated with sodium citrate (pH 5.5) at a final concentration of 50 mM, and incubated with Endo H (New England Biolabs) at 37 °C for 3 h. Alternatively, sodium phosphate (pH 7.5) and NP-40 were added to the denatured cell lysates at the final concentration of 50 mM and 1%, respectively, and the mixture was incubated with PNGase F (New England Biolabs) at 37 °C for 3 h. Immunoprecipitated and deglycosylated samples were boiled in SDS-PAGE sample buffer (62.5 mM Tris-HCl, pH 6.8; 2% SDS; 10% glycerol; and 0.1% bromophenol blue) containing β-ME, analyzed by SDS-PAGE, and visualized by autoradiography.

### BCR activation

Mouse B cells were suspended in serum-free RPMI medium supplemented with 25 mM HEPES, stimulated with F(ab’)2 fragments of goat anti-mouse IgM antibodies (20 μg/mL) (SouthernBiotech) for the indicated times, and lysed immediately by adding ice-cold lysis buffer (50 mM Tris-HCl, pH 8.0; 150 mM NaCl; 1% Triton X-100; and 1 mM EDTA) supplemented with protease inhibitor cocktail (Roche), 4 mM sodium pyrophosphate, 2 mM sodium vanadate and 10 mM sodium fluoride. Lysates were analyzed by SDS-PAGE and immunoblotted for molecules of interest using specific antibodies.

### Immunization

Mice were intraperitoneally injected with TNP_40_-AECM-Ficoll or TNP_0.5_-LPS (40 μg per mouse; LGC Biosearch Technologies) dissolved in PBS.

### Enzyme-linked immunosorbent assay (ELISA) and enzyme-linked immunospot (ELISPOT) assay

For the detection of TNP-specific antibodies by ELISA, plates were coated with TNP_33_-BSA. ELISA analyses of mouse IgM, IgG, IgA, IgE, and IgG3 in sera from unimmunized and immunized mice were achieved using HRP-conjugated secondary antibodies against each mouse antibody isotype (SouthernBiotech) and the 3,3′,5,5′-tetramethylbenzidine (TMB) liquid substrate system (Sigma). For ELISPOT assays, MultiScreen-IP filter plates (EMD Millipore) were coated with TNP_33_-BSA. Splenocytes and bone marrow cells were serially diluted across the plate and subsequently incubated for 16 h at 37 °C. HRP-conjugated goat anti-mouse IgM or IgG antibodies (SouthernBiotech) diluted in blocking buffer (10% FBS in PBS) were added. The spots were detected using TMB substrate for ELISPOT (Mabtech) and scanned and counted with an ImmunoSpot Analyzer (Cellular Technology Ltd.).

### Epitope mapping

Full-length human STING cDNA (template) and specific PCR primer sets were used to amplify STING DNA fragments encoding various truncated cytoplasmic domains of human STING (amino acids 139–379). These DNA fragments were subsequently subcloned into the pGEX-6P-1 vector. The constructs encoding various GST-truncated human STING fusion proteins were sequenced and induced to be expressed in BL21 (DE3) cells using isopropyl-β-d-thiogalactopyranoside (Sigma).

### Patient samples

Primary human CLL cells were obtained by Dr. Pinilla-Ibarz from patients diagnosed at the H. Lee Moffitt Cancer Center following the guidelines in the IRB protocol approved by the H. Lee Moffitt Cancer Center and the Wistar Institute and after informed consent was obtained from each patient in accordance with the Declaration of Helsinki.

### Statistics

To compare the percentages and numbers of cell populations among experimental groups, the data were graphed as the means ± SEM, and statistical significance (*p* < 0.05) was determined by unpaired two-tailed Student’s *t* test. Statistical significance for the ELISA data was determined by the Mann–Whitney *U* test.

### Online supplemental material

Figure S1 documents the generation of the STING V154M knock-in mouse model. Figure S2 documents the generation and epitope mapping of a mouse monoclonal antibody against STING and the generation of a polyclonal antibody against STING phosphorylated at S365. Figure S3 shows the gating strategies for analyses of B cells in the spleens. Figure S4 shows the gating strategies for analyses of B cells in the bone marrow and peripheral blood and plasma cells in the spleens and bone marrow. Figure S5 shows decreases in T cell populations in the spleens and peripheral blood but not in the bone marrow in unimmunized V154M mice compared to unimmunized WT mice. Figure S6 shows increases in the percentages of myeloid cells in the spleens, bone marrow and peripheral blood in unimmunized V154M mice compared to unimmunized WT mice. Figure S7 shows that purified B cells from V154M mice responded to LPS stimulation in culture by differentiating into antibody-secreting GL7+ or XBP1s+ plasmablasts that synthesized class I and class II MHC molecules and delivered them to the cell surface normally. Figure S8 shows that STING did not interact with the ER-resident calnexin chaperone or other type I transmembrane proteins in LPS-stimulated B cells. Figure S9 shows that proinflammatory cytokines did not affect antibody production, surface presentation of the BCR, or BCR signaling. Figure S10 documents the generation of the STING^flox/flox^ mouse model. Figure S11 shows that STING^KO^ B cells, plasmablasts and plasma cells displayed higher levels of the BCR on their surface than their STING-proficient counterparts. Figure S12 shows that LPS-stimulated plasmablasts from B cell-specific STING^KO^ mice exhibited significantly increased BCR signaling upon activation and that unimmunized B cell-specific STING^KO^ mice and their unimmunized WT littermates exhibited similar basal levels of anti-TNP IgM and IgG3 in the blood. Figure S13 shows that CLL cells could be purified from the spleens of CLL-bearing STING^WT^/Eµ-TCL1 and STING^KO^/Eµ-TCL1 mice and that STING^KO^/Eµ-TCL1 CLL cells expressed higher levels of IgM and Igβ on their surface than STING^WT^/Eµ-TCL1 CLL cells.

## Supplementary information

Supplementary legends and figures
