# Supplementary material for: STING regulates BCR signaling in normal and malignant B cells
Source: Cell Mol Immunol. 2020 Sep 30;18(4):1016–31. doi: 10.1038/s41423-020-00552-0 (PMC8115116; doi:10.1038/s41423-020-00552-0)
Supplement: Supplementary file 1 — Supplementary legends and figures [file 41423_2020_552_MOESM1_ESM.pdf]

STING regulates BCR signaling in normal and malignant B cells

Tang et al.

Table of contents: Page 1

Supplementary Figure Legends: Pages 2 - 8

Supplementary Figures S1-S13: Pages 9 - 21

Supplementary Figure Legends

Supplementary Figure 1. Confirmation of the long- and short-arm integration of the STING V154M targeting vector by Southern blots. Genomic DNA was extracted from 5 different clones carrying the Neo-deleted allele, and digested with Hpa I or Hind III, respectively. Southern blots were performed using a radiolabeled probe to confirm the integration of 3' and 5' homology arms. The expected band sizes of the WT allele and Neo-deleted allele after restriction digestion by Hpa I are 35010 bp and 16022 bp, respectively. The expected band sizes of the WT allele and Neo-deleted allele after restriction digestion by Hind III are 11256 bp and 8165 bp, respectively. Genomic DNA from WT mice was used as a control. The sequences of primers used to genotype mice carrying the Neo-deleted allele with V154M are listed beneath the blots.

Supplementary Figure 2. Epitope mapping of a mouse monoclonal antibody against STING and generation of a polyclonal antibody against STING phosphorylated at S365. (A) Mouse A20 cells, A20 cells treated with 20 µM 3'3'-cGAMP for 4 h, STING-deficient A20 STING-ZFN cells, and human H929 cells were immunoblotted with a newly generated mouse monoclonal anti-STING antibody (clone #370.142). (**B-C**) Lysates from transformed BL21 (DE3) cells expressing various GST-truncated human STING fusion proteins were analyzed on SDS-PAGE gels, and either stained with Coomassie Brilliant Blue G-250 (B) or immunoblotted with the anti-STING monoclonal antibody (C). The highest molecular weight bands recognized by the anti-STING antibody represent the intact fusion proteins; the lower molecular weight bands are degradation products. (**D**) Amino acid sequences of human and mouse STING epitopes (labeled in red) recognized by the anti-STING monoclonal antibody. Amino acids labeled in blue have similar chemical properties. (**E**) Amino acid sequences of the phospho-peptide and the backbone peptide of mouse STING used for generation and purification of the anti-phospho-S365 antibody.

Supplementary Figure 3. Gating strategies for analyses of B cells in the spleens. (**A**) Splenocytes from WT and V154M mice were stained with B220-BV605, GL7-PE, AA4.1-PE-Cy7, CD1d-PerCP-Cy5.5, and CD23-FITC. Gated B220+ B cells were analyzed for GL7+ activated B cells. Gated B220+/GL7-/AA4.1- B cell populations were analyzed for the CD1d+/CD23- marginal zone and CD1d-/CD23+ follicular B cells. The numbers of spleen cells from WT and V154M mice were counted using a hemocytometer. (**B-E**) Quantification of B220+ B cells (B), follicular B cells (C), marginal zone B cells (D), and GL7+ activated B cells (E) in spleens of unimmunized WT (n=10) and V154M (n=10) mice.

Supplementary Figure 4. Gating strategies for analyses of B cells in the bone marrow and peripheral blood and plasma cells in the spleens and bone marrow. (**A**) Bone marrow cells isolated from WT and V154M mice were stained with B220-BV605, CD43-PE, CD19-Alexa 647, IgM-PE-Cy7 and IgD-FITC. Gated B220+ total B cell populations (left panel) were analyzed for CD43+/CD19low pro-B cells and CD43+/CD19high pre-B cells (middle panel). The gated CD43-/CD19+ population (middle panel) were also analyzed for IgM+/IgD- immature B cells and IgM+/IgD+ mature B cells (right panel). The numbers of bone marrow cells from WT and V154M mice were counted using a hemocytometer. (**B-F**) Quantification of total B cell progenitors (B), pro-B cells (C), pre-B cells (D), immature B cells (E), and mature B cells (F) in the bone marrow of unimmunized WT (n=5) and V154M (n=5) mice. (**G**) Peripheral white blood cells from WT and V154M mice were stained with B220-Alexa 488. B220+ B cells were gated and analyzed. The numbers of white blood cells in the whole blood of WT and V154M mice were obtained using a HemaTrue Hematology Analyzer. (**H**) Quantification of B220+ B cells in the peripheral blood of unimmunized WT (n=5) and V154M (n=5) mice. (**I**) The spleen and bone marrow cells from unimmunized WT and V154M mice were stained with CD138-PE and XBP1s-Alexa 647. CD138+/XBP1s+ plasma cells were gated and analyzed.

Supplementary Figure 5. Decreased T cell populations in the spleens and peripheral blood but not in the bone marrow of unimmunized V154M mice. (**A**) Splenocytes from WT and V154M mice

were stained with CD3-APC-Cy7, B220-Alexa 488, CD4-BV605 and CD8-PE-Cy7. CD3+/B220- T cell populations were analyzed for CD4+ and CD8+ T cells. (**B-D**) Quantification of CD3+ T cells (B), CD4+ T cells (C), and CD8+ T cells (D) in the spleens of unimmunized WT (n=10) and V154M (n=10) mice. (**E**) Bone marrow cells from WT and V154M mice were stained with CD3-APC-Cy7, B220-Alexa 488, CD4-BV605 and CD8-PE-Cy7. CD3+/B220- T cell populations were analyzed for CD4+ and CD8+ T cells. (**F-H**) Quantification of CD3+ T cells (F), CD4+ T cells (G), and CD8+ T cells (H) in the bone marrow of unimmunized WT (n=15) and V154M (n=15) mice. (**I**) Peripheral white blood cells from WT and V154M mice were stained with CD3-APC-Cy7, B220-Alexa 488, CD4-BV605 and CD8-PE-Cy7. CD3+/B220- T cell populations were analyzed for CD4+ and CD8+ T cells. (**J-L**) Quantification of CD3+ T cells (J), CD4+ T cells (K), and CD8+ T cells (L) in the peripheral blood of unimmunized WT (n=15) and V154M (n=15) mice.

Supplementary Figure 6. Increased CD11b+/Ly6C+ monocytic cells and CD11b+/Ly6G+ granulocytic cells in the spleens, bone marrow and peripheral blood of unimmunized V154M mice. (**A**) Splenocytes from WT and V154M mice were stained with CD11c-BV421, CD11b-PE, Ly6C-Alexa 488 and Ly6G-Alexa 647. Gated CD11c-/CD11b+ myeloid populations were analyzed for Ly6C+/Ly6G- monocytic cells and Ly6C^{intermediate}/Ly6G+ granulocytic cells. (**B-C**) Quantification of CD11b+/Ly6C+ monocytic cells (B) and CD11b+/Ly6G+ granulocytic cells (C) in the spleens of unimmunized WT (n=10) and V154M (n=10) mice. (**D**) Bone marrow cells from WT and V154M mice were stained with CD11c-BV421, CD11b-PE, Ly6C-Alexa 488 and Ly6G-Alexa 647. Gated CD11c-/CD11b+ myeloid populations were analyzed for Ly6C+/Ly6G- monocytic cells and Ly6C^{intermediate}/Ly6G+ granulocytic cells. (**E-F**) Quantification of CD11b+/Ly6C+ monocytic cells (E) and CD11b+/Ly6G+ granulocytic cells (F) in the bone marrow of unimmunized WT (n=15) and V154M (n=15) mice. (**G**) Peripheral white blood cells from WT and V154M mice were stained with CD11c-BV421, CD11b-PE, Ly6C-Alexa 488 and Ly6G-Alexa 647. Gated CD11c-/CD11b+ myeloid populations were analyzed for Ly6C+/Ly6G- monocytic cells and Ly6C^{intermediate}/Ly6G+ granulocytic cells. (**H-I**)

Quantification of CD11b+/Ly6C+ monocytic cells (H) and CD11b+/Ly6G+ granulocytic cells (I) in the peripheral blood of unimmunized WT (n=15) and V154M (n=15) mice.

Supplementary Figure 7. Purified B cells from V154M mice could respond to LPS stimulation in culture by differentiating into antibody-secreting GL7+ or XBP1s+ plasmablasts which synthesized class I and class II MHC molecules and delivered them to the cell surface normally. (**A**) B cells purified from WT and V154M mice were stimulated with LPS for a course of 3 days. Each day, B cells were stained with B220-BV605 and GL7-PE. Gated B220+ B cells were analyzed for GL7+ populations. (**B**) Quantification (means ± SEM) of GL7+ plasmablasts (stimulated with LPS for 2 or 3 days) derived from WT (n=5) and V154M (n=5) mice. (**C**) B cells purified from WT and V154M mice were stimulated with LPS for 3 days, and stained with B220-BV605, CD138-PE, and XBP1s-Alexa 647. Gated B220+ B cells were analyzed for XBP1s+ populations. (**D-E**) Three-day LPS-stimulated plasmablasts from WT and V154M mice were starved in cysteine- and methionine-free media for 1 h, radiolabeled for 15 min, and chased for indicated times. Intracellular and extracellular IgM were immunoprecipitated from lysates (D) and culture media (E), respectively, using an anti-μ antibody. Immunoprecipitates were analyzed by SDS-PAGE and autoradiography. The asterisk denotes endo-H-resistant complex glycans. (**F-G**) Three-day LPS-stimulated plasmablasts from WT and V154M mice were starved in cysteine- and methionine-free media for 1 h, radiolabeled for 15 min, and chased for indicated times. Lysates were immunoprecipitated with an anti-class I MHC heavy chain antibody (F) or an anti-class II MHC α chain antibody (G). Immunoprecipitates were analyzed by SDS-PAGE and autoradiography. CHO and CHO* indicate high mannose-type glycans and complex-type glycans, respectively.

Supplementary Figure 8. STING did not interact with ER-resident calnexin chaperone or other type I transmembrane proteins in LPS-stimulated B cells. (**A**) Purified WT and V154M B cells were stimulated with LPS (20 μg/mL) for 3 days. Lysates were immunoblotted for indicated proteins. (**B**) B

cells purified from WT, V154M and STING-deficient mice were stimulated with LPS for 3 days, lysed in 1.25% digitonin lysis buffer, and analyzed by immunoblots for calnexin, class I MHC heavy chains, class II MHC α chains, and p97. Additionally, the anti-STING antibody was used to perform immunoprecipitations from the same lysates (equal protein amounts), and the immunoprecipitates were analyzed for the presence of calnexin, class I MHC heavy chains, class II MHC α chains, and p97 by immunoblots. (**C**) 5TGM1 and 5TGM1 STING-ZFN (STING-deficient) cells were lysed in 1.25% digitonin lysis buffer, and analyzed by immunoblots for calnexin and p97. In addition, the anti-STING antibody was used to perform immunoprecipitations from the same lysates (equal protein amounts), and the immunoprecipitates were analyzed for the presence of calnexin and p97.

Supplementary Figure 9. Pro-inflammatory cytokines did not affect antibody production, surface presentation of the BCR, and BCR signaling. (**A-B**) Two-day LPS-stimulated WT plasmablasts were treated with IFNβ (100 ng/mL) or TNFα (100 ng/mL) for another 24 h in the presence of LPS. Cells were subsequently starved in cysteine- and methionine-free media for 1 h, radiolabeled for 15 min, chased for indicated times, and lysed in RIPA buffer. IgM was immunoprecipitated from culture media (A), and Igβ was immunoprecipitated from lysates (B). Immunoprecipitates were analyzed by SDS-PAGE and autoradiography. CHO and CHO* indicate high mannose-type glycans and complex-type glycans, respectively. (**C**) Two-day LPS-stimulated WT plasmablasts were treated with IFNβ (100 ng/mL) or TNFα (100 ng/mL) for another 24 h in the presence of LPS. Cells were subsequently stimulated with goat anti-mouse IgM F(ab')2 (20 μg/mL) for indicated times, and lysed for immunoblot analyses of indicated proteins.

Supplementary Figure 10. Confirmation of the long- and short-arm integration of the STING$^{flox/flox}$ targeting vector by Southern blots. Genomic DNA was extracted from 5 different clones carrying the STING$^{flox/wt}$ targeted allele, and digested with Apa I or Afl II, respectively. Southern blots were performed using a radiolabeled probe to confirm the integration of 3' and 5' homology arms. The

expected band sizes of the WT allele and targeted allele after restriction digestion by Apa I are 33.5 Kb and 12407 bp, respectively. The expected band sizes of the WT allele and targeted allele after restriction digestion by Afl II are 3 Kb and 13700 bp, respectively. Genomic DNA from WT mice was used as a control. The sequences of primers used to genotype mice carrying the Neo-deleted allele are listed below the blot.

Supplementary Figure 11. STING^{KO} B cells, plasmablasts and plasma cells displayed increased levels of the BCR on their surface. (**A-B**) Freshly purified B cells from STING^{WT} and B cell-specific STING^{KO} mice were starved in cysteine- and methionine-free media for 1 h, radiolabeled for 15 min, and chased for a course of 2 h. Lysates were immunoprecipitated with an anti-μ heavy chain antibody (A) or an anti-Igβ antibody (B). Immunoprecipitated proteins were analyzed by SDS-PAGE and autoradiography. The asterisk denotes endo-H-resistant complex glycans. CHO and CHO* indicate high mannose-type glycans and complex-type glycans, respectively. (**C**) Densitometric quantitation of radiolabeled μ chains and Igβ at the 120-min time point in panels A and B was achieved using Phosphorimaging. (**D**) Freshly purified B cells from CD19Cre and B cell-specific STING^{KO} (CD19Cre/STING$^{flox/flox}$) mice were stimulated with LPS (20 μg/mL) for a course of 3 days. Each day, cells were surface stained with B220-BV605 and IgM-PE-Cy7. Gated B220+ populations were analyzed for the expression of IgM. This experiment is independent of that performed for quantification presented in Fig. 7A. (**E**) CD19Cre and B cell-specific STING^{KO} mice were intraperitoneally immunized with TNP-Ficoll on Day 0. On Day 9, bone marrow cells from immunized mice were stained with B220-Alexa 488, CD138-PE and Igβ-APC. Gated B220-/CD138+ plasma cells were analyzed for the expression of Igβ.

Supplementary Figure 12. LPS-stimulated plasmablasts from B cell-specific STING^{KO} mice exhibited significantly increased BCR signaling upon activation; in addition, B cell-specific STING^{KO} mice and their WT littermates exhibited similar basal levels of anti-TNP IgM and IgG3 in the blood. (**A**) 1-day LPS-stimulated and (**B**) 2-day LPS-stimulated plasmablasts from STING^{WT} and

STING^{KO} mice were stimulated with goat anti-mouse IgM F(ab')2 (20 μg/mL) for indicated times, and lysed for immunoblot analyses. (**C-D**) Serum levels of anti-TNP IgM (C) and IgG3 (D) in unimmunized STING^{WT} (n=9) and B cell-specific STING^{KO} (n=6) mice were determined by ELISA.

Supplementary Figure 13. Purification of CLL cells from spleens of CLL-bearing STING^{WT}/Eμ-TCL1 and STING^{KO}/Eμ-TCL1 mice; in addition, STING^{KO}/Eμ-TCL1 CLL cells expressed higher levels of IgM and Igβ than STING^{WT}/Eμ-TCL1 CLL cells on their surface. (**A**) Splenocytes from STING^{WT}/Eμ-TCL1 and STING^{KO}/Eμ-TCL1 mice were treated with RBC lysis buffer. Some cells were subjected to purification of CD19+B220^{low}CD5+ CLL cells using Pan B cell isolation kit (Miltenyi Biotec). Cells before and after purification were stained with CD3-BV605, CD19-APC-Cy7, B220-Alexa 488 and CD5-APC. Gated CD3-/CD19+ B cell populations were analyzed for B220^{high}/CD5- precancerous B cell populations and B220^{low}CD5+ CLL populations. (**B**) Spleen cells from STING^{WT}/Eμ-TCL1 and STING^{KO}/Eμ-TCL1 mice were surface stained with CD19-APC-Cy7, IgM-Alexa 568, B220-Alexa 488 and CD5-APC. Gated CD19+ B cell populations were analyzed for B220^{low}CD5+ CLL populations. Gated CLL populations were analyzed for the expression of IgM. (**C**) Spleen cells from STING^{WT}/Eμ-TCL1 and STING^{KO}/Eμ-TCL1 mice were surface stained with CD19-APC-Cy7, Igβ-FITC, B220-BV605 and CD5-APC. Gated CD19+ B cell populations were analyzed for B220^{low}CD5+ CLL populations. Gated CLL populations were analyzed for the expression of Igβ.

Figure S1

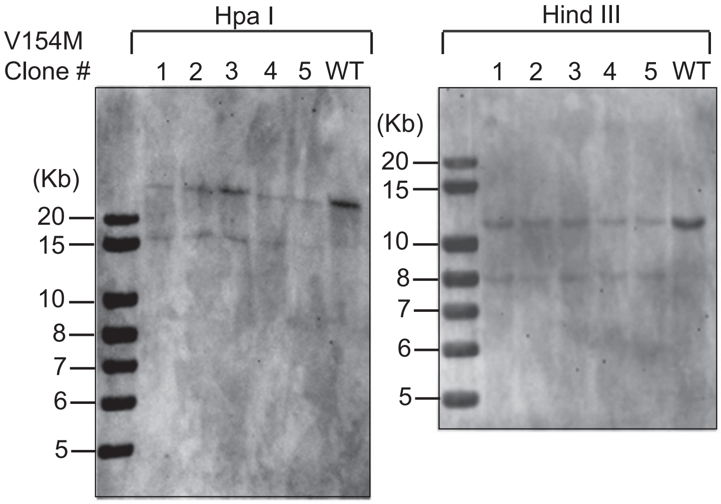

Genotyping primers:
Forward: 5’-ACA ACT TTG CTT CTA TAG GTA TCT CGC-3’
Reverse: 5‘-ATG AGT TCC CCA GCA GAG TGG-3’

Figure S2

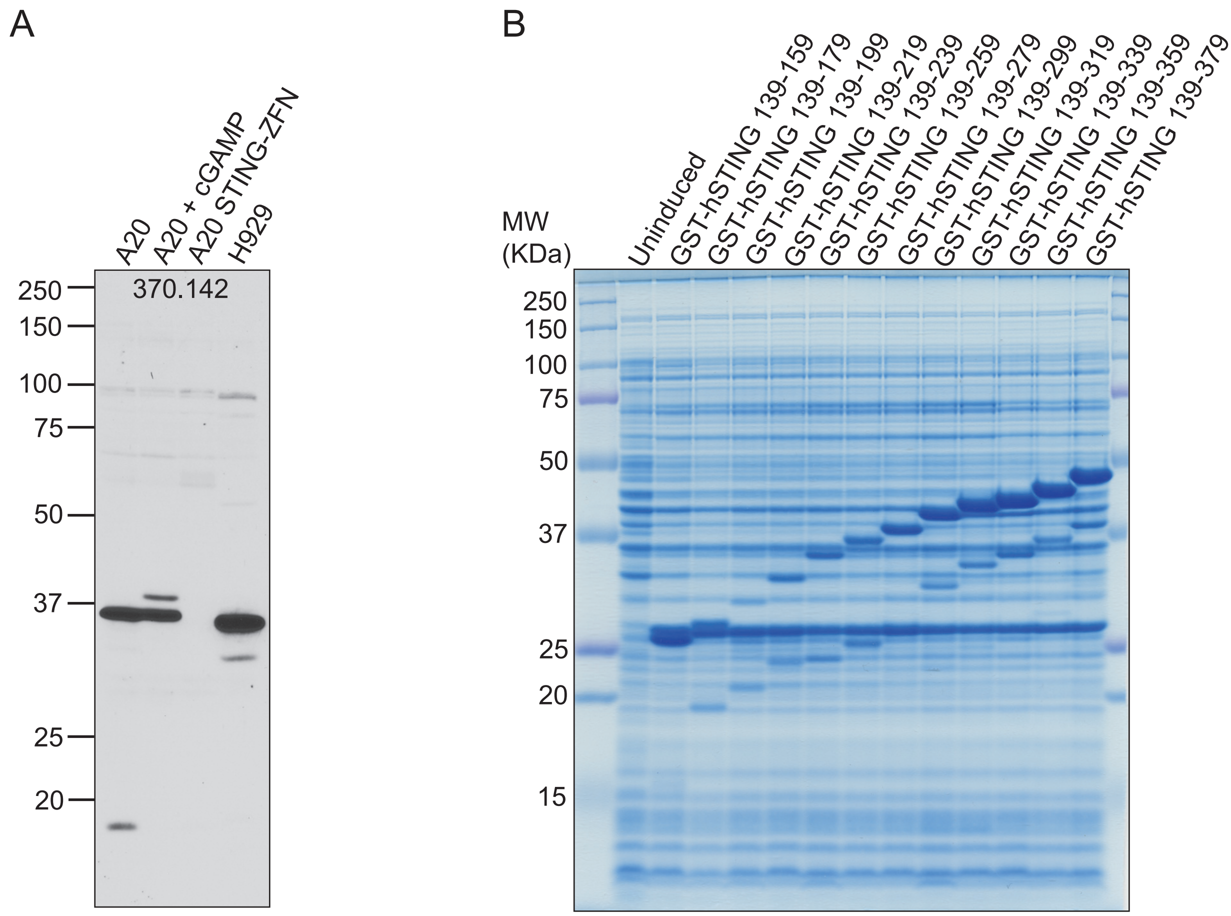

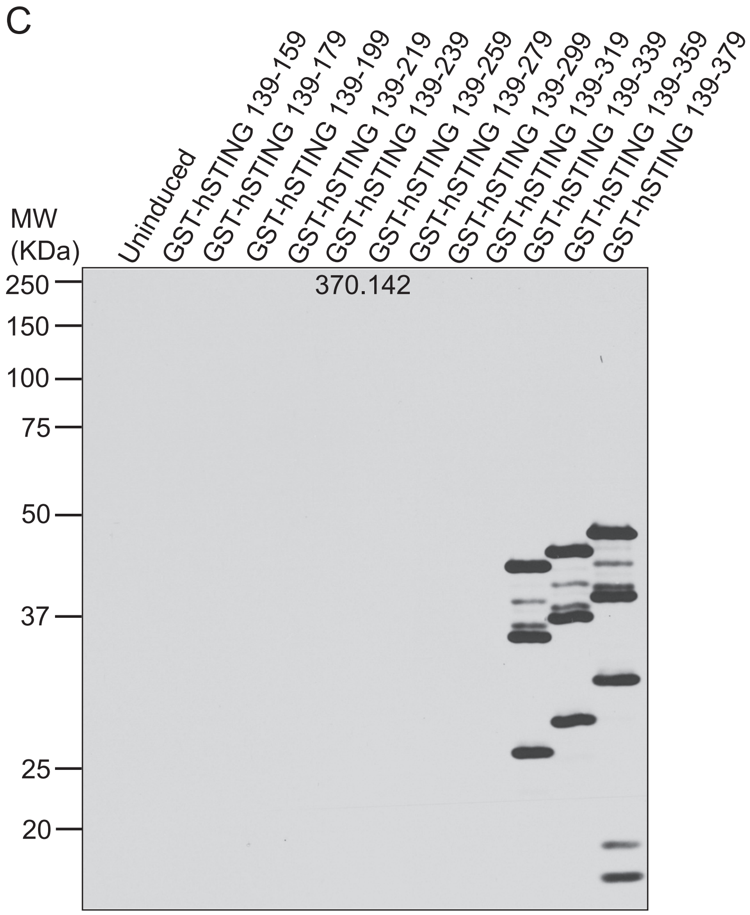

D

Human STING epitope sequence
319-DDSSFSLSQEVLRHLRQEEKE-339
Mouse STING epitope sequence
318-DGNSFSLSQEVLRHIRQEEKE-338

E

Phospho-peptide sequence
(for immunization and positive affinity purification):
Cys-EPRLLI(pS)GMDQPLP
Backbone peptide sequence
(for negative affinity purification):
Cys-EPRLLISGMDQPLP

Figure S3

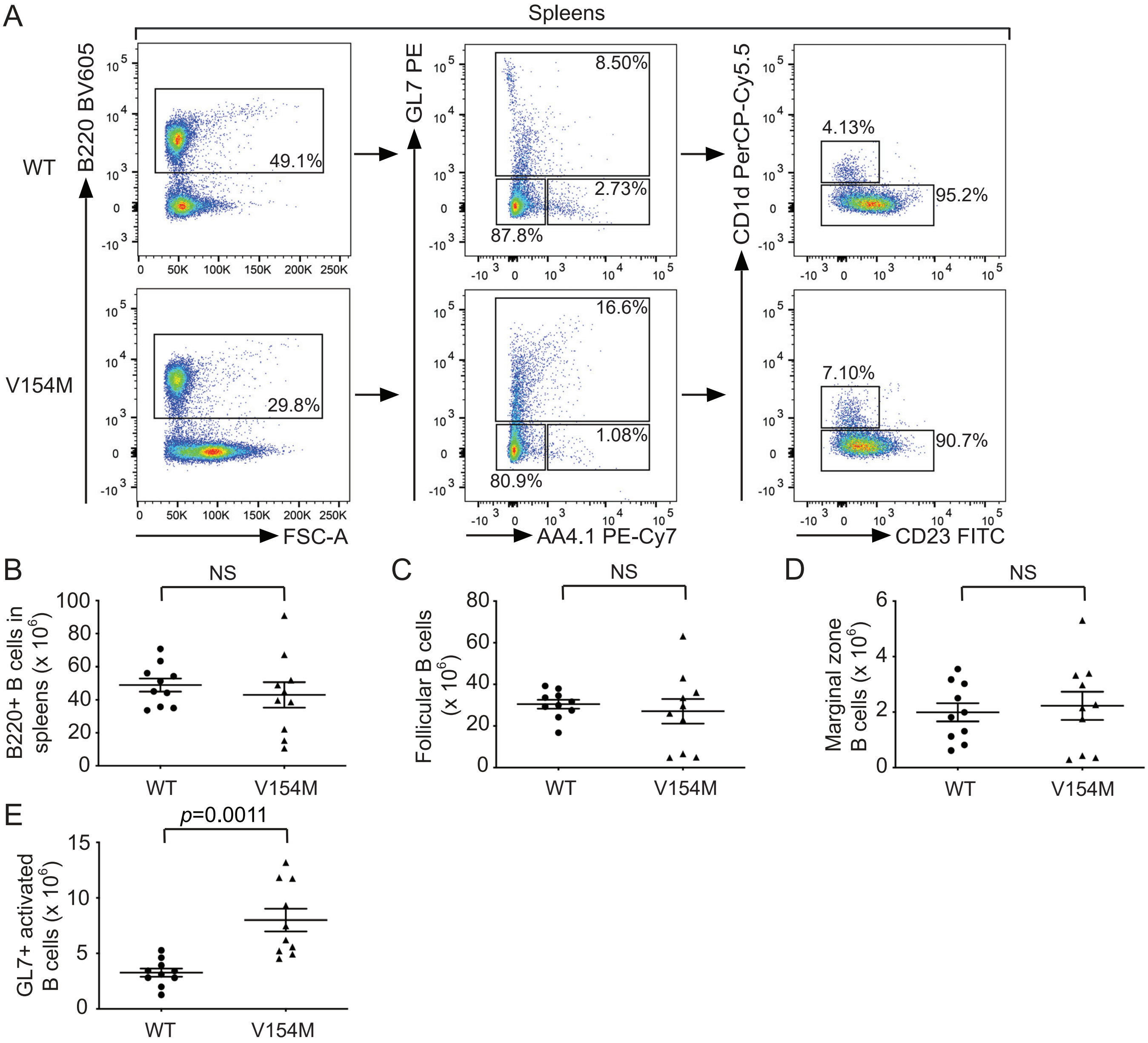
A
Spleens
WT
V154M
B220 BV605
49.1%
29.8%
FSC-A
GL7 PE
8.50%
2.73%
87.8%
16.6%
1.08%
80.9%
AA4.1 PE-Cy7
CD1d PerCP-Cy5.5
4.13%
95.2%
7.10%
90.7%
CD23 FITC
B
NS
B220+ B cells in spleens (x 10^6)
WT
V154M
C
NS
Follicular B cells (x 10^6)
WT
V154M
D
NS
Marginal zone B cells (x 10^6)
WT
V154M
E
p=0.0011
GL7+ activated B cells (x 10^6)
WT
V154M

Figure S4

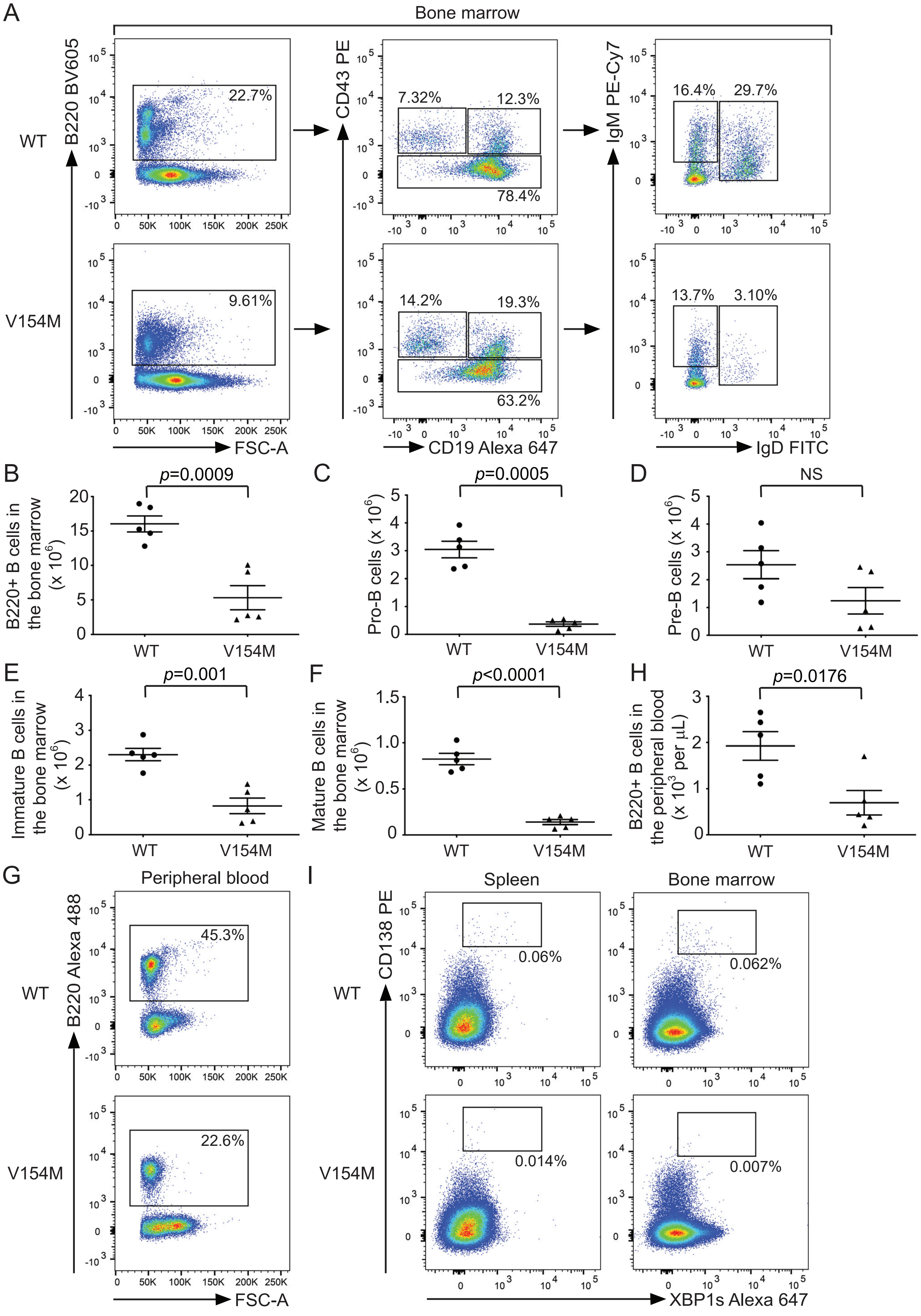
A
Bone marrow
WT
V154M
B220 BV605
FSC-A
22.7%
9.61%
CD43 PE
CD19 Alexa 647
7.32%
12.3%
78.4%
14.2%
19.3%
63.2%
IgM PE-Cy7
IgD FITC
16.4%
29.7%
13.7%
3.10%
B
p=0.0009
B220+ B cells in the bone marrow (x 10^6)
C
p=0.0005
Pro-B cells (x 10^6)
D
NS
Pre-B cells (x 10^6)
E
p=0.001
Immature B cells in the bone marrow (x 10^6)
F
p<0.0001
Mature B cells in the bone marrow (x 10^6)
H
p=0.0176
B220+ B cells in the peripheral blood (x 10^3 per µL)
G
Peripheral blood
B220 Alexa 488
45.3%
22.6%
I
Spleen
Bone marrow
CD138 PE
XBP1s Alexa 647
0.06%
0.062%
0.014%
0.007%

Figure S5

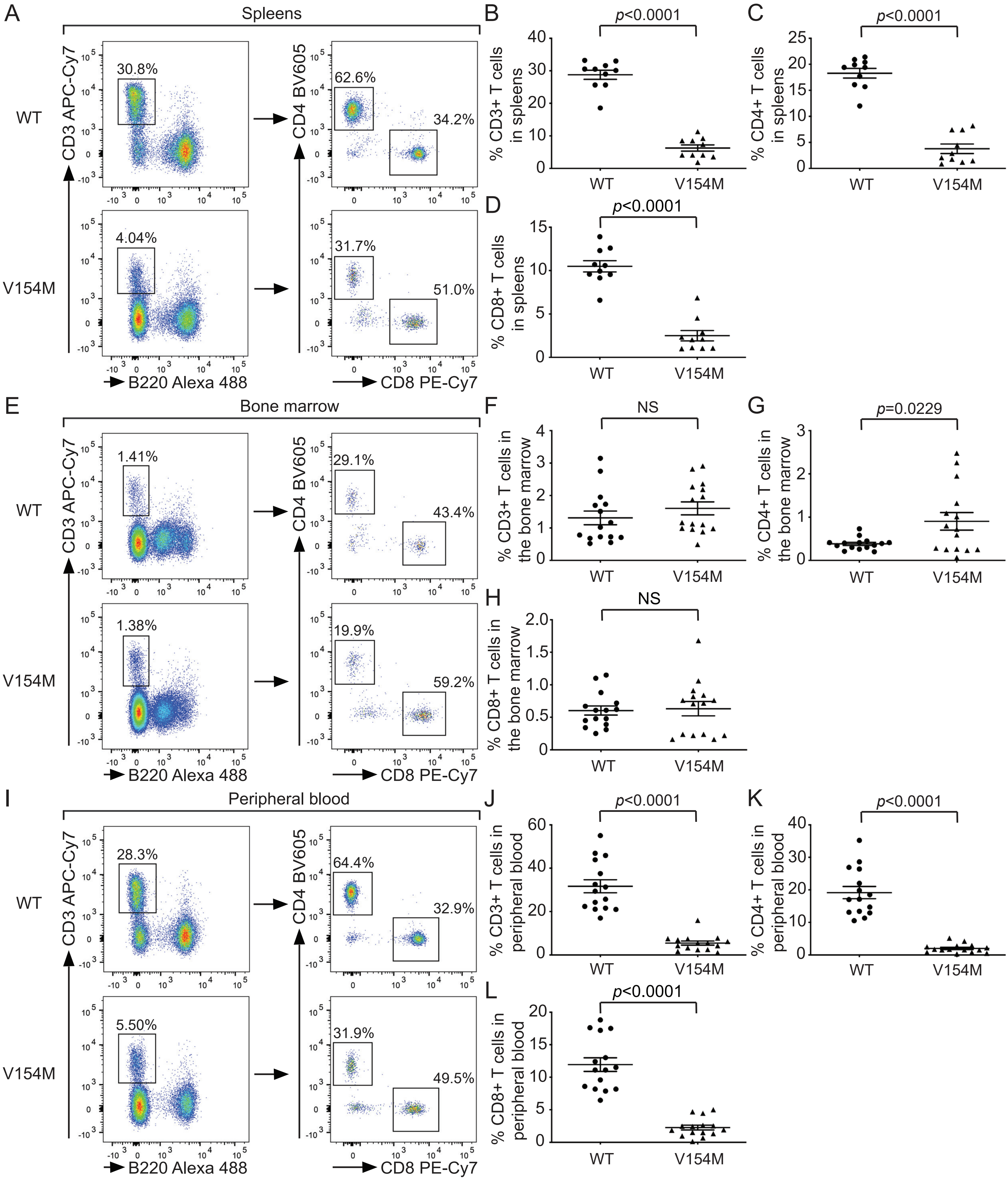
A
Spleens
WT
V154M
30.8%
4.04%
62.6%
34.2%
31.7%
51.0%
CD3 APC-Cy7
B220 Alexa 488
CD4 BV605
CD8 PE-Cy7
B
p<0.0001
% CD3+ T cells in spleens
WT
V154M
C
p<0.0001
% CD4+ T cells in spleens
D
p<0.0001
% CD8+ T cells in spleens
E
Bone marrow
1.41%
1.38%
29.1%
43.4%
19.9%
59.2%
F
NS
% CD3+ T cells in the bone marrow
G
p=0.0229
% CD4+ T cells in the bone marrow
H
NS
% CD8+ T cells in the bone marrow
I
Peripheral blood
28.3%
5.50%
64.4%
32.9%
31.9%
49.5%
J
p<0.0001
% CD3+ T cells in peripheral blood
K
p<0.0001
% CD4+ T cells in peripheral blood
L
p<0.0001
% CD8+ T cells in peripheral blood

Figure S6

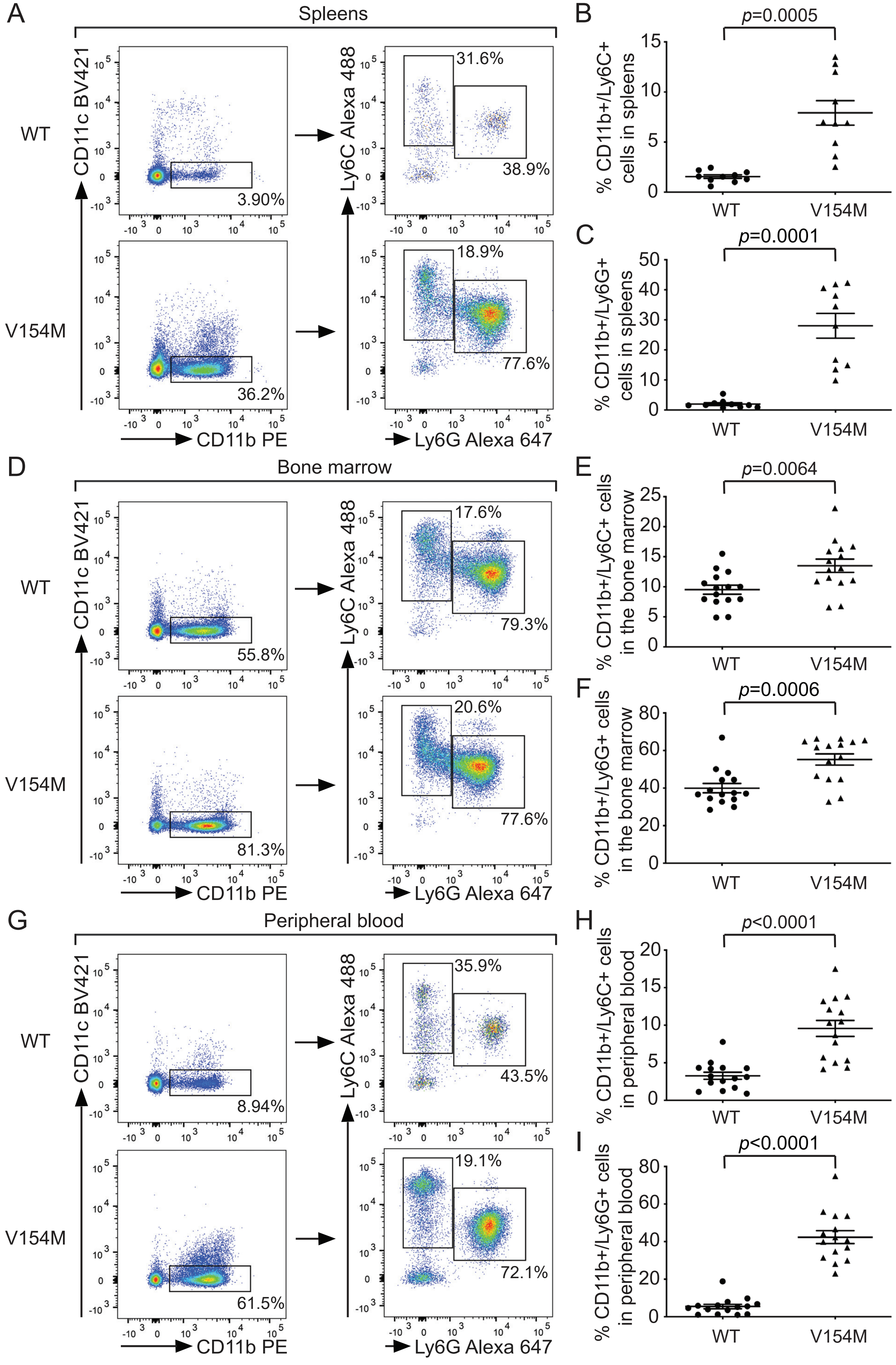

A
Spleens
WT
V154M
CD11c BV421
CD11b PE
Ly6C Alexa 488
Ly6G Alexa 647
3.90%
31.6%
38.9%
36.2%
18.9%
77.6%
B
p=0.0005
% CD11b+/Ly6C+ cells in spleens
C
p=0.0001
% CD11b+/Ly6G+ cells in spleens
D
Bone marrow
55.8%
17.6%
79.3%
81.3%
20.6%
77.6%
E
p=0.0064
% CD11b+/Ly6C+ cells in the bone marrow
F
p=0.0006
% CD11b+/Ly6G+ cells in the bone marrow
G
Peripheral blood
8.94%
35.9%
43.5%
61.5%
19.1%
72.1%
H
p<0.0001
% CD11b+/Ly6C+ cells in peripheral blood
I
p<0.0001
% CD11b+/Ly6G+ cells in peripheral blood

Figure S7

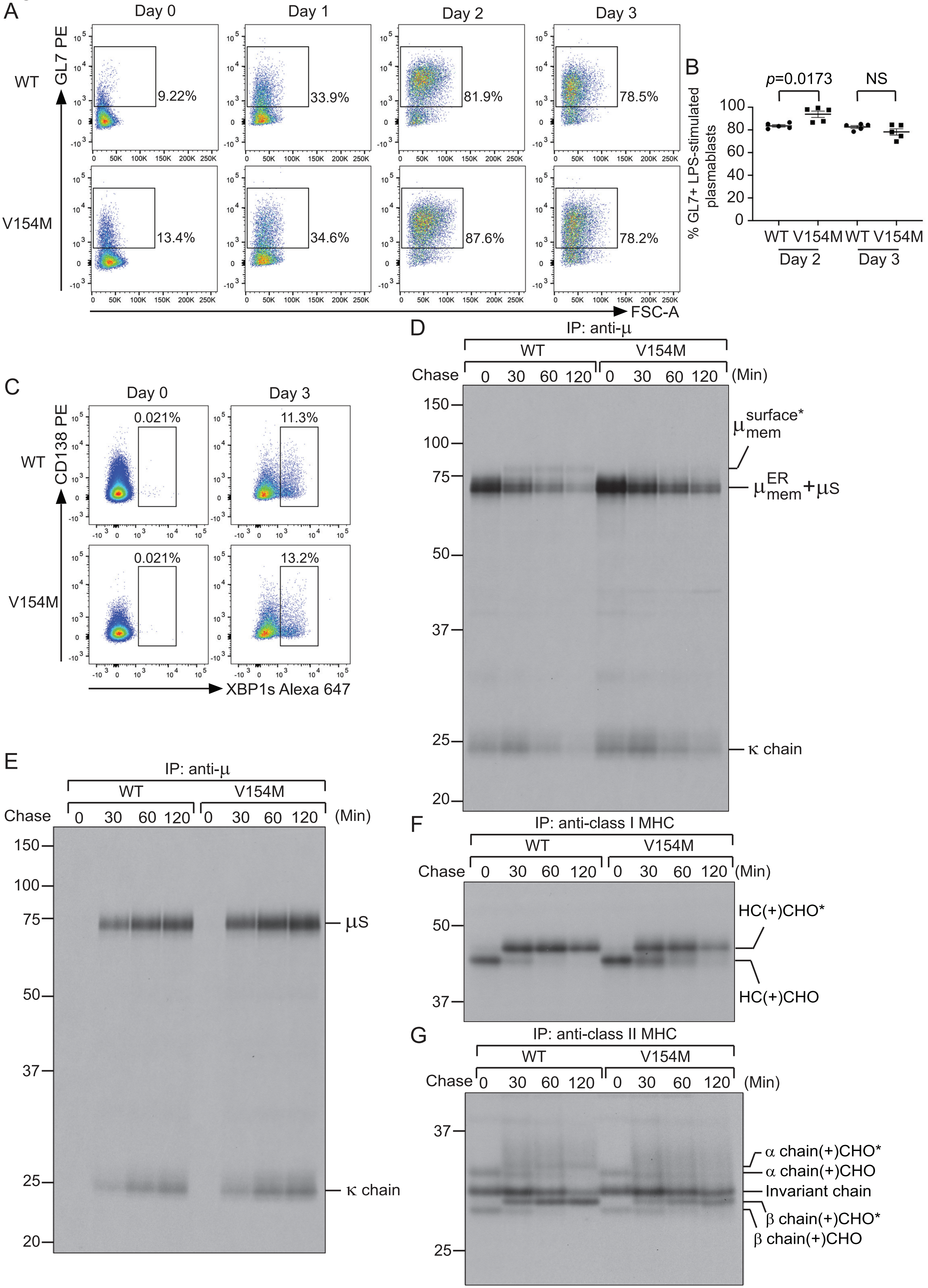
A
Day 0
Day 1
Day 2
Day 3
GL7 PE
WT
V154M
9.22%
33.9%
81.9%
78.5%
13.4%
34.6%
87.6%
78.2%
FSC-A
B
p=0.0173
NS
% GL7+ LPS-stimulated plasmablasts
WT V154M WT V154M
Day 2
Day 3
C
Day 0
Day 3
CD138 PE
WT
V154M
0.021%
11.3%
0.021%
13.2%
XBP1s Alexa 647
D
IP: anti-μ
WT
V154M
Chase 0 30 60 120 0 30 60 120 (Min)
μ surface* mem
μ ER mem + μS
κ chain
E
IP: anti-μ
WT
V154M
Chase 0 30 60 120 0 30 60 120 (Min)
μS
κ chain
F
IP: anti-class I MHC
WT
V154M
Chase 0 30 60 120 0 30 60 120 (Min)
HC(+)CHO*
HC(+)CHO
G
IP: anti-class II MHC
WT
V154M
Chase 0 30 60 120 0 30 60 120 (Min)
α chain(+)CHO*
α chain(+)CHO
Invariant chain
β chain(+)CHO*
β chain(+)CHO

Figure S8

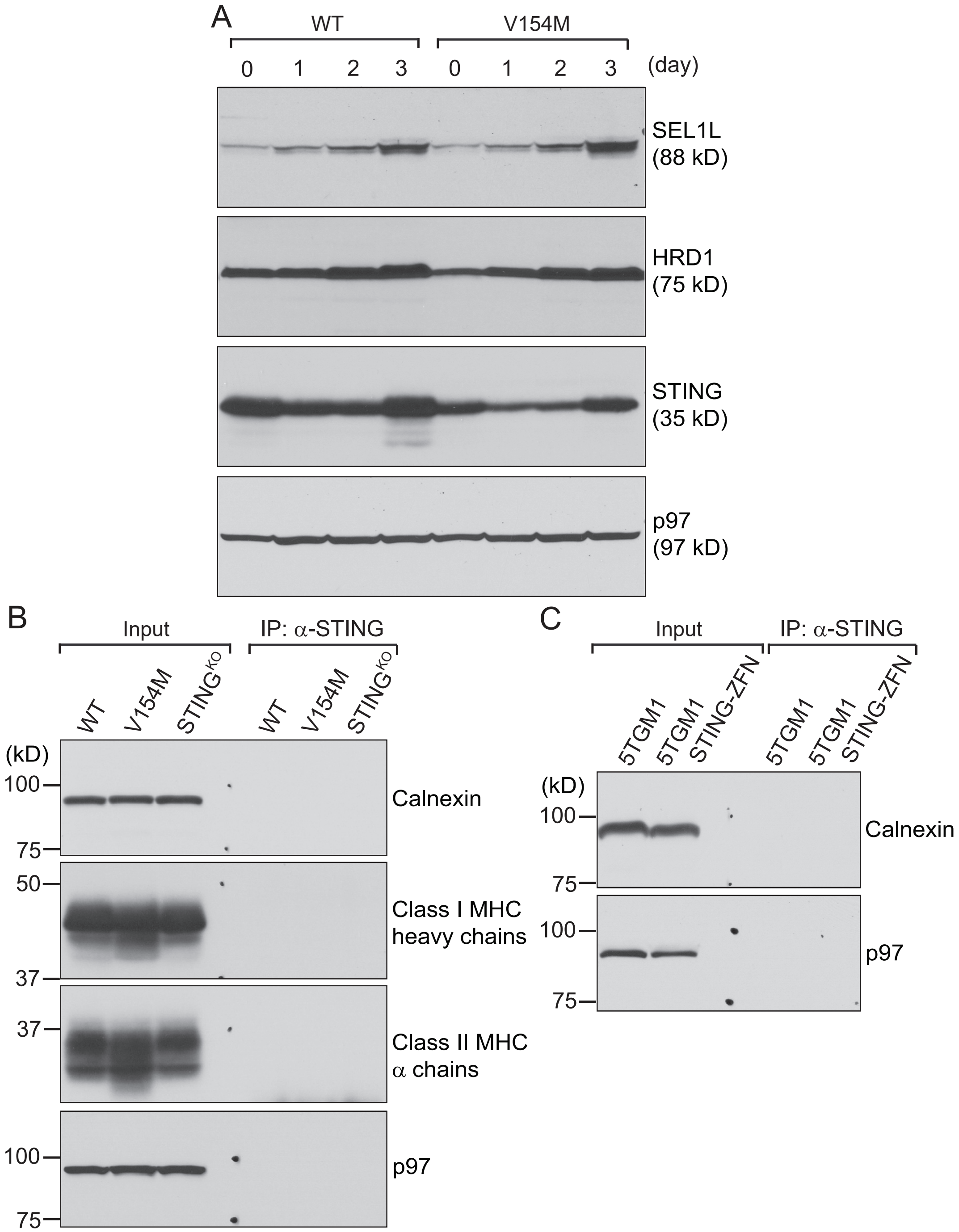

A
WT
V154M
0 1 2 3 0 1 2 3 (day)
SEL1L
(88 kD)
HRD1
(75 kD)
STING
(35 kD)
p97
(97 kD)
B
Input
IP: α-STING
WT
V154M
STING^KO
WT
V154M
STING^KO
(kD)
100
75
50
37
37
100
75
Calnexin
Class I MHC
heavy chains
Class II MHC
α chains
p97
C
Input
IP: α-STING
5TGM1
5TGM1 STING-ZFN
5TGM1
5TGM1 STING-ZFN
(kD)
100
75
100
75
Calnexin
p97

Figure S9

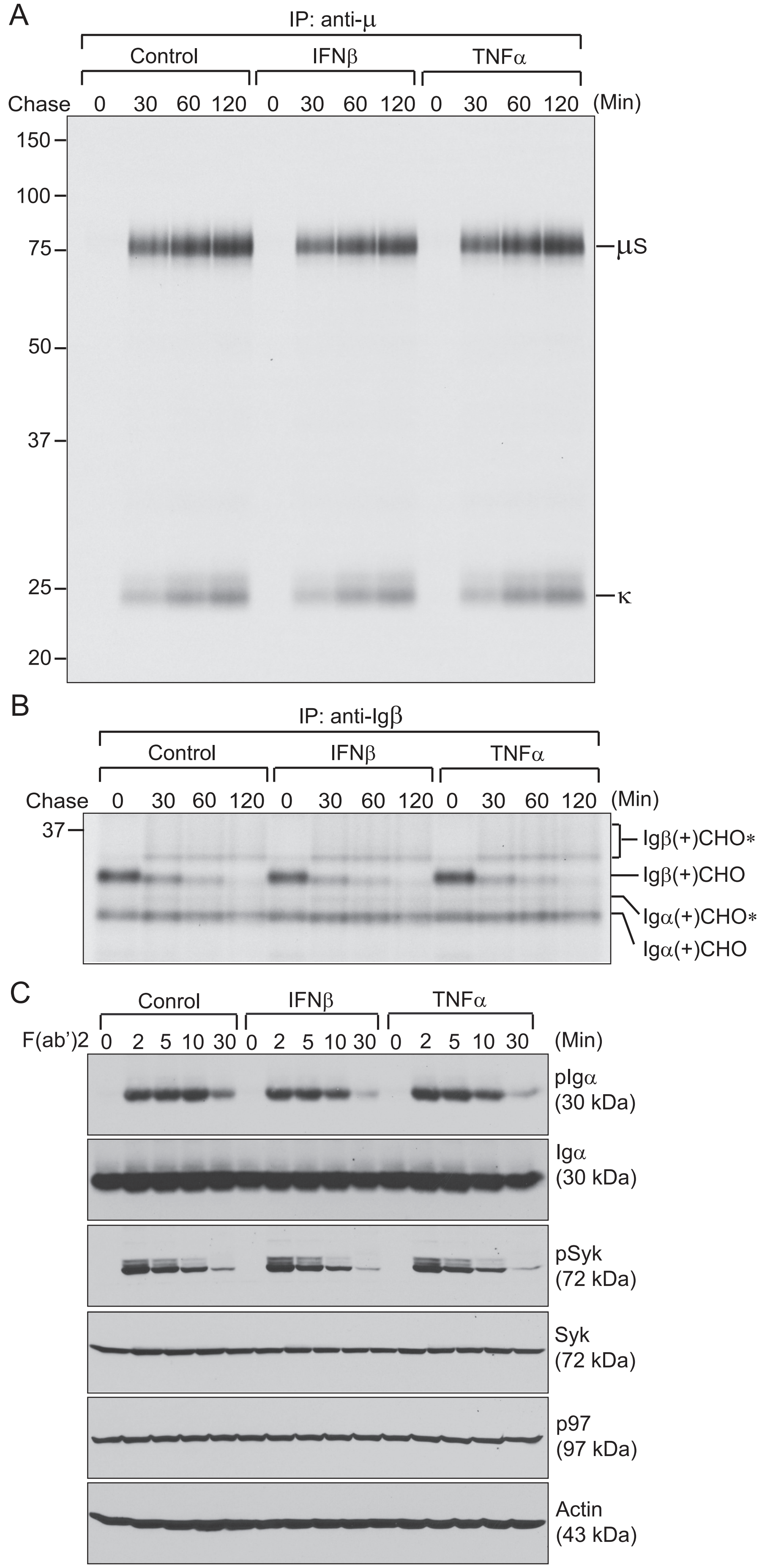

A
IP: anti-μ
Control
IFNβ
TNFα
Chase 0 30 60 120 0 30 60 120 0 30 60 120 (Min)
150
100
75
50
37
25
20
μS
κ
B
IP: anti-Igβ
Control
IFNβ
TNFα
Chase 0 30 60 120 0 30 60 120 0 30 60 120 (Min)
37
Igβ(+)CHO*
Igβ(+)CHO
Igα(+)CHO*
Igα(+)CHO
C
Conrol
IFNβ
TNFα
F(ab')2 0 2 5 10 30 0 2 5 10 30 0 2 5 10 30 (Min)
pIgα (30 kDa)
Igα (30 kDa)
pSyk (72 kDa)
Syk (72 kDa)
p97 (97 kDa)
Actin (43 kDa)

Figure S10

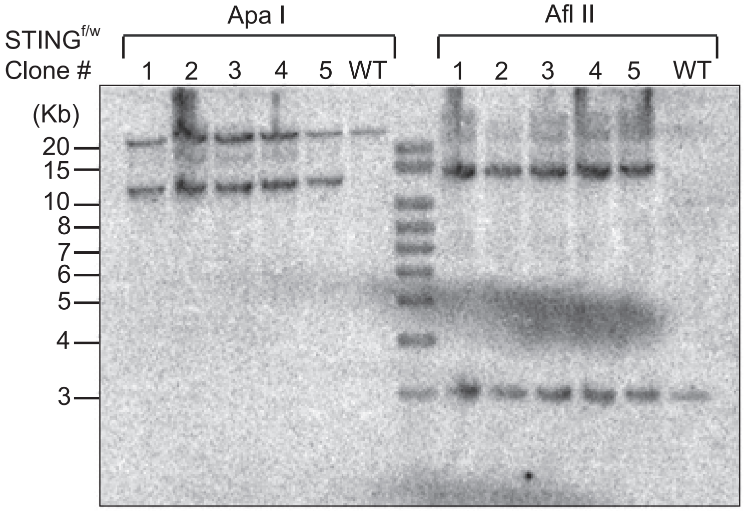

Genotyping primers:
Forward: 5’-CAC AGC TTT GCC GTA CTT ATT TTG C-3’
Reverse: 5‘-TGC CTC AAA GAT CAC ACC CTT CAC-3’

Figure S11

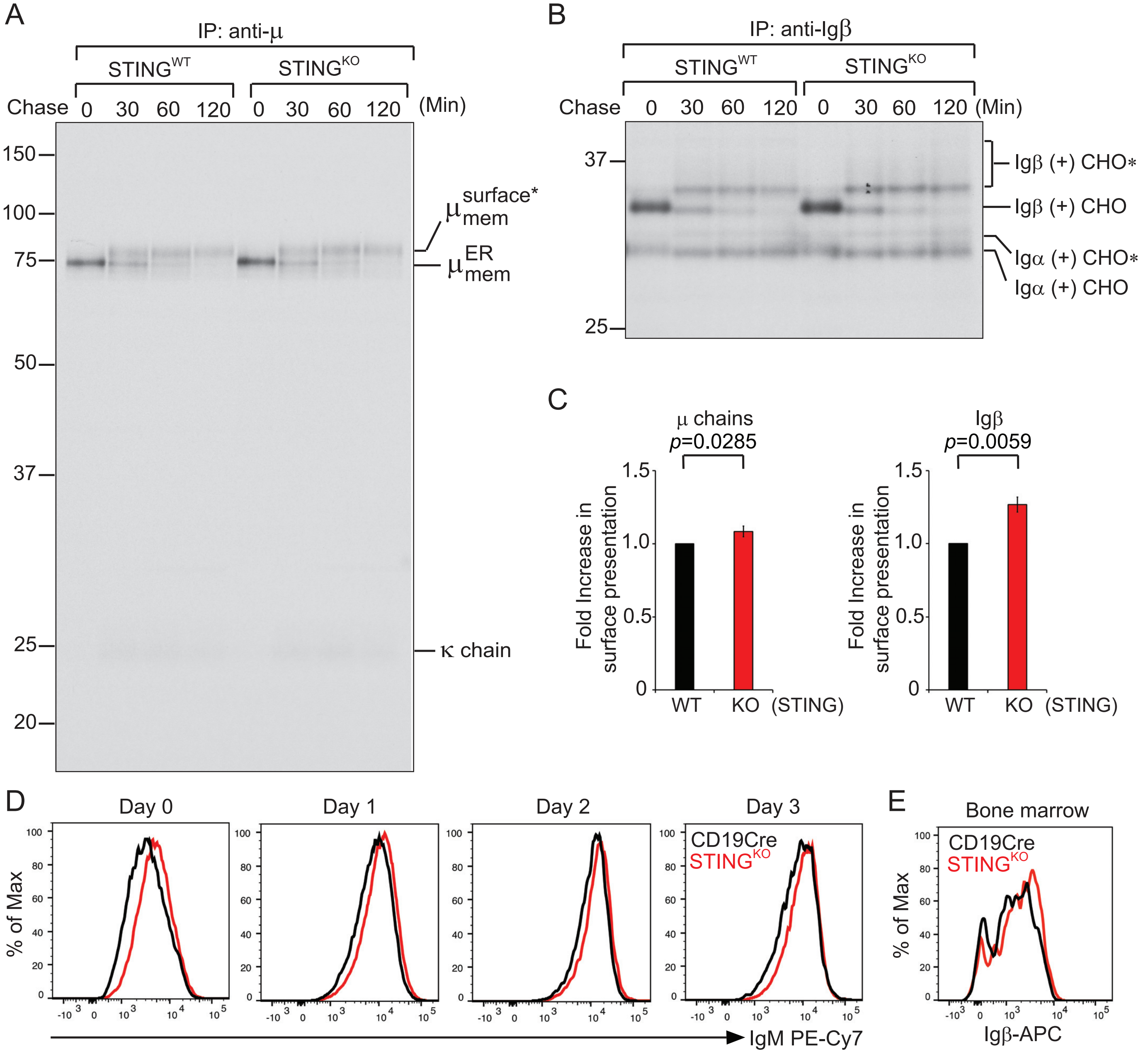

A
IP: anti-μ
STING^WT
STING^KO
Chase 0 30 60 120 0 30 60 120 (Min)
150
100
75
50
37
25
20
μ surface* mem
μ ER mem
κ chain
B
IP: anti-Igβ
STING^WT
STING^KO
Chase 0 30 60 120 0 30 60 120 (Min)
37
25
Igβ (+) CHO*
Igβ (+) CHO
Igα (+) CHO*
Igα (+) CHO
C
μ chains
p=0.0285
Igβ
p=0.0059
Fold Increase in surface presentation
1.5
1.0
0.5
0
WT KO (STING)
D
Day 0
Day 1
Day 2
Day 3
% of Max
CD19Cre
STING^KO
IgM PE-Cy7
E
Bone marrow
CD19Cre
STING^KO
% of Max
Igβ-APC

Figure S12

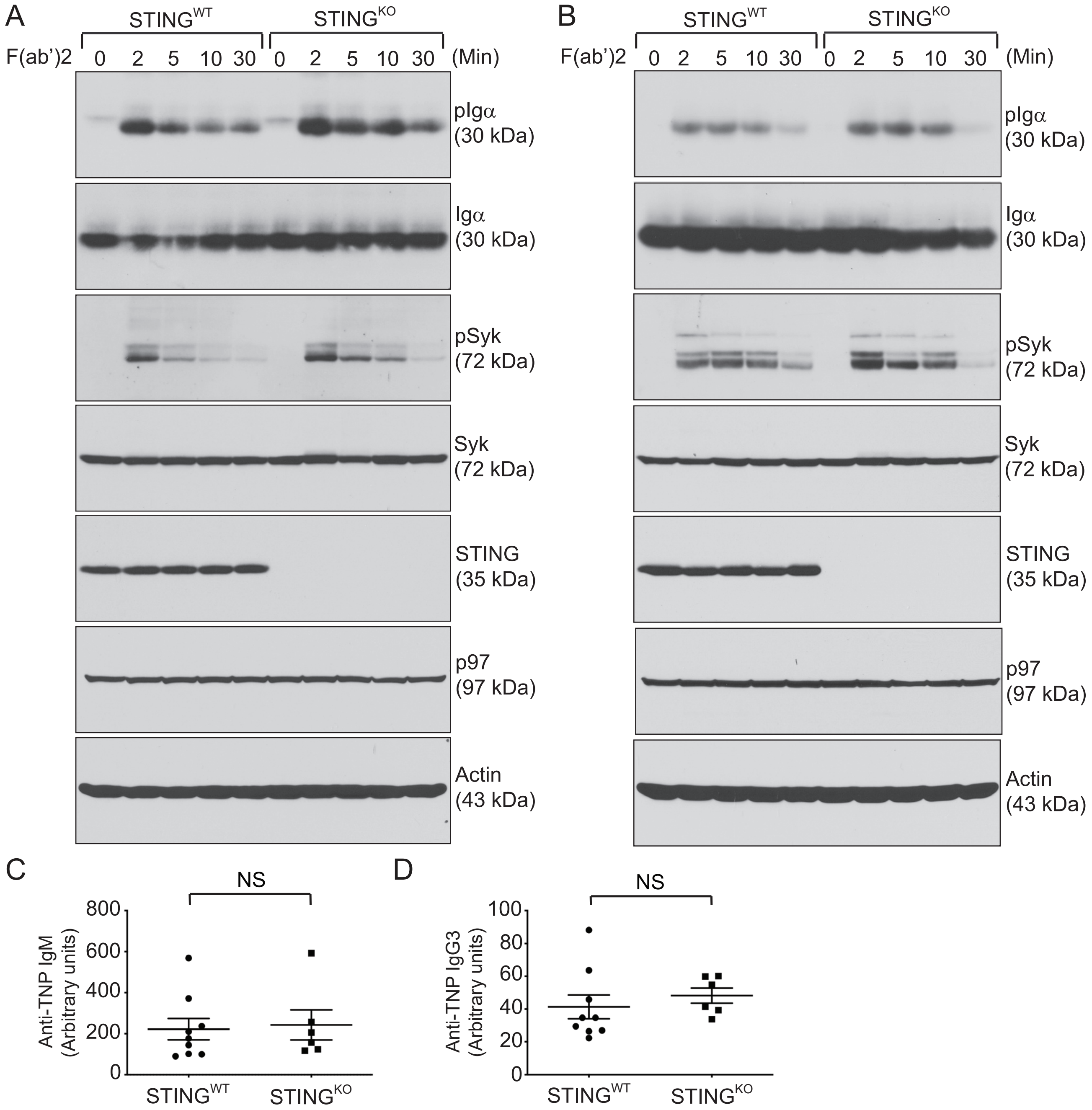
A
B
STING^WT
STING^KO
F(ab')2 0 2 5 10 30 0 2 5 10 30 (Min)
pIgα (30 kDa)
Igα (30 kDa)
pSyk (72 kDa)
Syk (72 kDa)
STING (35 kDa)
p97 (97 kDa)
Actin (43 kDa)
C
NS
Anti-TNP IgM (Arbitrary units)
0
200
400
600
800
STING^WT
STING^KO
D
NS
Anti-TNP IgG3 (Arbitrary units)
0
20
40
60
80
100
STING^WT
STING^KO

Figure S13

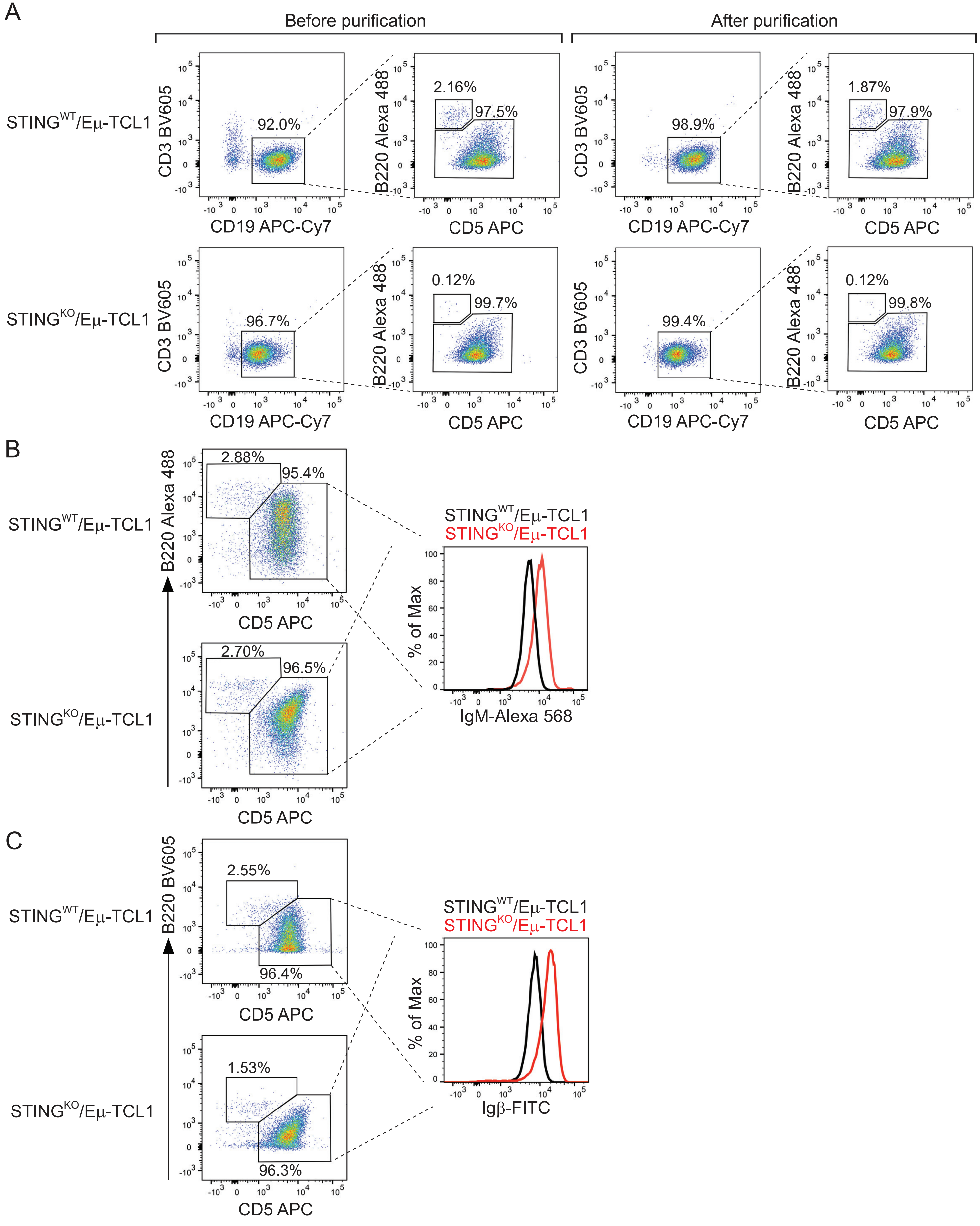
A
Before purification
After purification
STING^WT/Eμ-TCL1
STING^KO/Eμ-TCL1
CD3 BV605
CD19 APC-Cy7
B220 Alexa 488
CD5 APC
92.0%
2.16%
97.5%
98.9%
1.87%
97.9%
96.7%
0.12%
99.7%
99.4%
0.12%
99.8%
B
2.88%
95.4%
2.70%
96.5%
STING^WT/Eμ-TCL1
STING^KO/Eμ-TCL1
% of Max
IgM-Alexa 568
C
B220 BV605
2.55%
96.4%
1.53%
96.3%
Igβ-FITC
